# Structural insights into the PrpTA toxin–antitoxin system in *Pseudoalteromonas rubra*

**DOI:** 10.3389/fmicb.2022.1053255

**Published:** 2022-11-24

**Authors:** Chenchen Wang, Chuanying Niu, Khan Muhammad Hidayatullah, Lu Xue, Zhongliang Zhu, Liwen Niu

**Affiliations:** ^1^MOE Key Laboratory for Membraneless Organelles and Cellular Dynamics, School of Life Sciences, Division of Life Sciences and Medicine, University of Science and Technology of China, Hefei, China; ^2^Center for Infection and Immunity, Guangzhou Institutes of Biomedicine and Health, Chinese Academy of Sciences, Guangzhou, China

**Keywords:** PrpTA, toxin–antitoxin system, MDT, conformational changes, protein oligomerization, plasmid replication, RHH

## Abstract

Bacteria could survive stresses by a poorly understood mechanism that contributes to the emergence of bacterial persisters exhibiting multidrug tolerance (MDT). Recently, *Pseudoalteromonas rubra prpAT* module was found to encode a toxin PrpT and corresponding cognate antidote PrpA. In this study, we first reported multiple individual and complex structures of PrpA and PrpT, which uncovered the high-resolution three-dimensional structure of the PrpT:PrpA^2^:PrpT heterotetramer with the aid of size exclusion chromatography-multi-angle light scattering experiments (SEC-MALS). PrpT:PrpA^2^:PrpT is composed of a PrpA homodimer and two PrpT monomers which are relatively isolated from each other and from ParE family. The superposition of antitoxin monomer structures from these structures highlighted the flexible C-terminal domain (CTD). A striking conformational change in the CTDs of PrpA homodimer depolymerized from homotetramer was provoked upon PrpT binding, which accounts for the unique PrpT-PrpA^RHH^ mutual interactions and further neutralizes the toxin PrpT. PrpA^2–54^-form I and II crystal structures both contain a doughnut-shaped hexadecamer formed by eight homodimers organized in a cogwheel-like form *via* inter-dimer interface dominated by salt bridges and hydrogen bonds. Moreover, PrpA tends to exist in solution as a homodimer other than a homotetramer (SEC-MALS) in the absence of flexible CTD. Multiple multi-dimers, tetramer and hexamer included, of PrpA^2–54^ mediated by the symmetric homodimer interface and the complicated inter-dimer interface could be observed in the solution. SEC-MALS assays highlighted that phosphate buffer (PB) and the increase in the concentration appear to be favorable for the PrpA^2–54^ oligomerization in the solution. Taken together with previous research, a model of PrpA^2–54^ homotetramer in complex with *prpAT* promoter and the improved mechanism underlying how PrpTA controls the plasmid replication were proposed here.

## Introduction

Drug-resistant2 bacterial pathogens, such as *Mycobacterium tuberculosis*, *Cryptococcus neoformans*, and *Cryptococcus gattii*, are of significant concern in recent years (Huemer et al., 2020; Tharappel et al., 2022). Multidrug tolerance (MDT) is directly caused by a small fraction of phenotypically variant subpopulation-persister cells, which are substantially dormant and characterized by slow growth rates, high expression of stationary phase markers, reduced protein synthesis, and low DNA replication (Schumacher et al., 2012, 2015; Page and Peti, 2016; Paul et al., 2019; Ma et al., 2021; Xia et al., 2021). MDT provides the basis for the subsequent rapid evolution of resistance and is thought to be the prerequisite for drug resistance and the primary reason for ineradicable bacterial and chronic infections (Fleming, 1929; Cohen et al., 2013; Maisonneuve and Gerdes, 2014; Lee and Lee, 2016; Levin-Reisman et al., 2017; Xie et al., 2018; Paul et al., 2019; Ma et al., 2021). Under endogenous or exogenous stress, the toxin–antitoxin system (TAS) could simultaneously mediate and accelerate the development of persister cells and MDT. Upon the removal of stress, persisters will resuscitate and restore normal growth leading to clinical recurrent infections, especially those caused by biofilms, and subsequent treatment failures (Cohen et al., 2013; Paul et al., 2019; Zhou et al., 2021b).

Toxin–antitoxin modules were initially identified as a plasmid stability factor in the conjugative plasmids in the 1980s (Ogura and Hiraga, 1983; Jaffe et al., 1985; Gerdes et al., 1986; Cooper and Heinemann, 2000; Van Melderen, 2010; Ni et al., 2021). Later, they turned out to be widely distributed on the chromosomes and mobile genetic elements (MGEs) in bacteria, archaea, and bacteriophage, especially pathogenic bacteria (Ogura and Hiraga, 1983; Hayes and Van Melderen, 2011; Fraikin et al., 2020; Leroux et al., 2020; Kamruzzaman et al., 2021; Ni et al., 2021; Xue et al., 2022). It is worth noting that MGE is closely related to the genetic stability and formation of persisters through horizontal transfer and vertical transmission (Cooper and Heinemann, 2000; Costa et al., 2001; Schumacher et al., 2012, 2015; Blair et al., 2015; Levin-Reisman et al., 2017; Ni et al., 2021; Xia et al., 2021). However, there is an ongoing controversy concerning the direct link between TAS persistence, and it is believed that at least not all TASs are necessarily related to persistence (Kim and Wood, 2016; Edelmann et al., 2020; Song and Wood, 2020). TAS, directly or indirectly, participates in the regulation of intracellular physiological activities including bacteriophage resistance, antibiotic tolerance, biofilm formation, and response to oxidative stress (Paul et al., 2019, 2022; Bertelsen et al., 2021; Kamruzzaman et al., 2021; Li et al., 2021; Ma et al., 2021; Qi et al., 2021; Xia et al., 2021; Du et al., 2022). Followed by the degradation of the corresponding antitoxin partner, the active toxin component was, thus, released to interact with cellular pathways to activate potentially deleterious toxic activities (Muthuramalingam et al., 2016; Leroux et al., 2020; Sarpong and Murphy, 2021; Xia et al., 2021). Furthermore, the type II TAS, which is studied the most and best, is the most abundant in bacterial MGEs among eight TAS types investigated to date (Xie et al., 2018; Kamruzzaman et al., 2021; Sarpong and Murphy, 2021; Zhang et al., 2021; Xue et al., 2022). For instance, *Ep*RatAB and *Ep*YefM-YoeB TAS from *Edwardsiella piscicida* are thought to be the model organism for the study of intracellular infections and refer to antibiotic resistance and host infection (Ma et al., 2021; Du et al., 2022).

A binary type II TAS consists of a stable metabolic inhibitor—toxin and corresponding labile antitoxin (Muthuramalingam et al., 2016; Leroux et al., 2020; Song and Wood, 2020; De Bruyn et al., 2021; Srivastava et al., 2021; Xia et al., 2021; Zhang et al., 2021; Jurenas et al., 2022). As for the antitoxin serving as a repressor, it is comprised of the N-terminal DNA-binding domain and the C-terminal toxin-neutralizing domain (Bobay et al., 2005; Coles et al., 2005; Xue et al., 2022). Taken together with the strand β 1, the helix–turn–helix (HTH) motif is supposed to be expanded into a ribbon–helix–helix (RHH) motif, as found in CopG/MetJ/Arc repressor superfamily which includes the plasmid transcriptional repressor prototype *Sa*CopG (PDB: 1EA4) from *Streptococcus agalactiae*, methionine repressor protein *Ec*MetJ (PDB: 1MJK) from *Escherichia coli*, and Arc repressor from *Salmonella* virus P22 (PDB: 1PAR) (Raumann et al., 1994; Garvie and Phillips, 2000; Costa et al., 2001; Schreiter and Drennan, 2007). RHH superfamily transcription factors (TFs) are of physiological importance toward the recognition between human pathogens and hosts (Schreiter and Drennan, 2007). RHH superfamily TFs always dock into the major grooves of duplex nucleic acid as a multi-dimer and further transcriptionally autoregulate (Knight and Sauer, 1989; Raumann et al., 1994; Schreiter and Drennan, 2007; Schumacher et al., 2015; Garcia-Rodriguez et al., 2021b). Even though some of them are dimeric in solution, they will instantly build up contacts in the corresponding DNA-bound complex with the exception of TraY whose polypeptide has two repeats of the RHH motif (Raumann et al., 1994; Costa et al., 2001; Solar et al., 2002; Schreiter and Drennan, 2007).

Recently, PrpTA was found to be a pMBL6842-encoded type II TAS with the unique function to regulate the plasmid replication in *Pseudoalteromonas rubra*. It consists of stable PrpT toxin and labile PrpA antitoxin canonically positioned adjacently within the same operon (Li et al., 2016; Ni et al., 2021). So far, there are few reports referring to the structural details of type II TA systems in marine bacteria. In this study, we reported for the first time the high-resolution structure of the PrpT:PrpA^2^:PrpT heterotetramer and two forms of crystal structures of truncated antitoxin-PrpA^2–54^. Moreover, a series of size exclusion chromatography-multi-angle light scattering experiments (SEC-MALS) assays were performed to investigate the oligomeric states of the PrpTA system. Our results reflected that the α 3 helixes of PrpA will bend obviously toward the PrpA^RHH^ domains of homodimer depolymerized from homotetramer upon PrpT binding, which consequently leads to the extra mutual interactions between toxin PrpT and N-terminal PrpA^RHH^ domains. PrpT together with PrpA finally forms a stable PrpT:PrpA^2^:PrpT heterotetramer, thus restricting the flexibility of PrpA^CTD^ and neutralizing the toxin PrpT. Furthermore, PrpA^2–54^-form I and II crystallize as a doughnut-shaped hexadecamer formed by eight vicinal homodimers *via* an inter-dimer interface dominated by salt bridges and hydrogen bonds. The incomplete hexadecameric rings, tetramer and hexamer included, could be observed in the solution presumably due to the unstructured C-terminal domain (CTD) that is absent in the crystallized entity like *V. cholerae* ParD2. It seems that the oligomerization of PrpA^2–54^ is partially affected by protein concentration and solution conditions. Moreover, a knowledge-based model for PrpA^2–54^ tetramer-*prpAT* promoter was proposed and discussed here. Overall, the PrpTA system assembly mechanism, antitoxin PrpA oligomerization, and the structural details of the mechanism underlying how PrpTA TAS controls plasmid replication could be further elucidated or improved.

## Materials and methods

### Protein expression and purification

All vectors were transformed into Rosetta (DE3) competent cells using the heat shock method. A single colony was inoculated in Luria-Bertani (LB) medium supplemented with the corresponding antibiotic, such as ampicillin (100 μg/ml) and kanamycin (50 μg/ml) at 310.15 K (37°C) and 220 rpm. Protein expression was induced with 1 mM Isopropyl β-D-1-thiogalactopyranoside (IPTG) once the bacterial growth reached OD600 of 0.8 and further incubated at 289.15 K (16°C) for 20 h. Bacterial cells were harvested by centrifugation at 8,000 rpm for 6 min and 277.15 K (4°C), followed by resuspension in 0.3 M NaCl and 0.05 M Tris pH 8.0, and further lysed with ultrasonicator (Qsonica; USA) in an ice bath. After centrifugation at 12,000 rpm for 30 min and 277.15 K (4°C), the protein was purified from the supernatant utilizing Ni-NTA resin (GE Healthcare: USA), followed by Superdex 75 pg column (HiLoad™ 16/600; GE Healthcare; USA). The purity and concentration of the proteins were assessed with SDS-PAGE and OneDrop™ OD-1000 + spectrophotometer (WINS; China), respectively. In addition, antitoxin PrpA^FL^ (expressed by vector PrpA-N-his-pET28a) carries a 6 × his tag on the N-terminus after being translated, and PrpA^254^ (expressed by vector PrpA^2–54^-C-his- pET28a) carries a 6 × his tag on the C-terminus. Toxin PrpT [expressed by vector PrpT-pET22b(+)] carries no tag. Their recombinant sequences could be accessed in Supplementary Table 1. The theoretical relative molecular weight of PrpT, PrpA^FL^ with N-terhexahistidine, and PrpA^2–54^ with C-terhexahistidine is ∼11.4, ∼11.66, and ∼7.05 kDa, respectively. Moreover, all vectors and oligonucleotide fragments used in the current study were obtained from Sangon Biotech Co., Ltd (Shanghai, China).

### Crystallization

The finely purified PrpA^2–54^ and PrpTA were concentrated to ∼5.2 and ∼3.6 mg/ml, respectively, in 0.1 M NaCl with 0.05 M Tris (pH 8.0). Initial crystal screening was performed by mixing an equal volume of protein and reservoir solution utilizing the sitting drop vapor diffusion method at 289.15 K (16°C). Two days post-crystallization, the crystal of PrpA^2–54^-form I was observed in a condition containing 0.2 M sodium acetate trihydrate, 0.1 M sodium citrate (pH 5.5), and 5% (w/v) PEG 4000. Similarly, crystals for PrpA^2–54^-form II were obtained in 0.2 M magnesium chloride hexahydrate, and crystals for PrpTA complex were obtained in 0.1 M HEPES sodium at pH 7.5, 30% (v/v) PEG 400, 0.1 M sodium cacodylate (pH 6.0), and 15% (w/v) PEG 4000.

### Data collection and structure determination

Crystals were picked with nylon loops and cryoprotected in a reservoir solution supplemented with 20% glycerol. All the diffraction datasets were collected at the beamlines in Shanghai Synchrotron Radiation Facility (SSRF) utilizing the single-wavelength small-angle oscillation method. All datasets were initially indexed and integrated with XDS, followed by scaling with an aimless module integrated into CCP4i (v7.1). The initial structural model of PrpA^2–54^ was determined by Phaser in PHENIX (v1.19.2) with *Vc*ParD2 antitoxin (PDB: 7B22) as a search template utilizing molecular replacement method, followed by model building with Autobuild module in PHENIX. Similarly, the structure of the PrpTA complex was searched against the refined structure of the PrpA^2–54^ monomer and *Caulobacter vibrioides* ParE toxin monomer. The structures were iteratively refined using Refmac5, WinCoot (v0.9.6), and phenix.refine offered by PHENIX. The detailed statistical information about these datasets is summarized in Supplementary Table 2.

### SEC-multi-angle light scattering experiments

SEC-multi-angle light scattering experiments were conducted using an AKTA™ pure HPLC system (GE Healthcare, USA) connected in-line with a DAWN HELEOS II (Wyatt Technology, Santa Barbara, CA, USA) eight-angle light-scattering detector, followed by a refractive-index detector (Wyatt Technology, Santa Barbara, CA, USA). SEC-MALS system was equilibrated with the corresponding running buffer at 0.5 mL/min for 12 h prior to the sample loading. A series of PrpA, PrpA^2–54^, and PrpTA samples were prepared in several kinds of buffers (including 50 mmol/L tris–HCl and 100 mmol/L NaCl pH 8.0, 50 mmol/L tris–HCl and a300 mmol/L NaCl pH 8.0, 50 mmol/L tris–HCl and 500 mmol/L NaCl pH 8.0, 50 mmol/L MES and 500 mmol/L NaCl pH 5.5, and 100 Mm (NaH^2^PO^4^/Na^2^HPO^4^) pH 8.0), and 0.1 ml was injected into the loop for each dilution. Accordingly, the absolute molar mass of each protein sample could be determined based on the data processed by the ASTRA (v7.0.1) offered by Wyatt company.

### Circular-dichroism spectroscopy

Circular-dichroism spectra were recorded at room temperature using a Chirascan™ qCD Spectrometer (Applied Photophysics Limited, UK) at a concentration of 0.4 mg/ml in 100 Mm (NaH^2^PO^4^/Na^2^HPO^4^) pH 8.0 based on 0.5 mm optical path. For each CD experiment, the background was measured one time, the buffer was measured two times, and samples were measured three times to reduce error and noise. All data were acquired with the subsequent parameters, bandwidth: 1 nm, wavelength scanning range: 180–260 nm, and time per point: 0.5. Pro-Data (v4.5.1825.0), and BeStSel were used to view and process CD spectra data to consequently access information on secondary structure composition.

### Modeling for PrpA^2–54^ tetramer complex with prpAT promoter

Sequence-specific interactions between antitoxin PrpA and duplex oligonucleotide are mostly mediated by N-terminal RHH domains of PrpA *via* recognizing the conserved 5’-(G/A)TTTG(T/A)AAT(A/G)-3’ motif which could be pseudo-palindromic or asymmetric (Ni et al., 2021). Based on the structure of transcriptional repressor CopG in complex with 22 bp dsDNA [PDB: 1EA4, which revealed a tetramer consisting of two dimers and associated by a crystallographic dyad, interacting in the same way as two dimers in the unliganded structure (Del Solar et al., 1989; Gomis-Ruth et al., 1998; Costa et al., 2001)], a possible dsDNA-RHH interaction mode was generated by NUCBIND. Combined with the structure of PrpA^2–54^ antitoxin homotetramer, a knowledge-based model of PrpA^2–54^ homotetramer in complex with *prpAT* promoter was proposed and later improved by energy minimization refinement and water refinement successively using refinement module offered by HADDOCK 2.4 with default parameters. The iteratively refined model was finally validated by the PISA server.

## Results

### PrpTA complex exists as a heterotetramer in solution and contains a PrpA homodimer depolymerized from homotetramer

**FIGURE 1:**
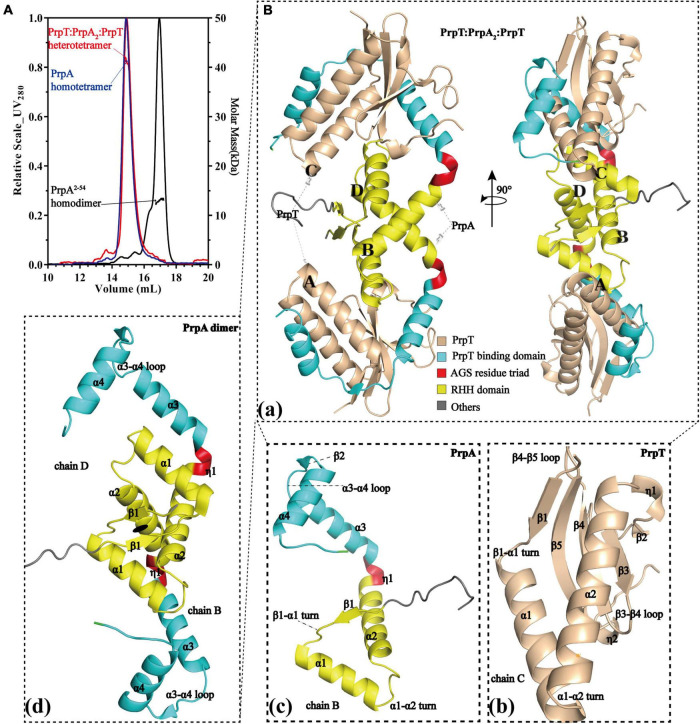
PrpT-PrpA^2^-PrpT heterotetramer consists of a PrpA homodimer and two PrpT monomers isolated from each other. **(A)** PrpTA complex (red), PrpA (blue), and PrpA^2–54^ (black) present an estimated absolute molecular weight matching tetramer, tetramer, and dimer, respectively. **(B)** Two views of PrpTA heterotetramer cartoon presentation **(a)**. Cartoon representations of PrpT toxin monomer **(b)**, PrpA^FL^ antitoxin monomer **(c)**, and PrpA^FL^ homodimer **(d)** (The black solid ellipse represents the local twofold axis, and all secondary structure elements are defined by DSSP plugin in PyMol).

To investigate the assembly mechanism of the PrpTA system in solution, a series of SEC-MALS experiments were performed on purified PrpTA complex, PrpA^FL^, and PrpA^2–54^ samples in this study (Figure 1A and Supplementary Figures 1, 2). Moreover, we succeeded in crystallizing PrpTA but failed to crystallize PrpA^FL^ due to the flexibility in the CTD reported before (Ni et al., 2021). However, we were lucky to crystallize truncated PrpA^FL^ (PrpA^2–54^) and finally solved two forms of PrpA^2–54^ crystal structures and the high-resolution PrpTA crystal structure (Figure 1B and Supplementary Figure 3). Considering the theoretical relative molecular weight of PrpT (∼11.4 kDa) and PrpA (∼11.66 kDa), our SEC-MALS results reflected that PrpTA complex, PrpA^FL^ and PrpA^2–54^ present an estimated absolute mass matching tetramer, tetramer and dimer, respectively. These results highlighted that deletion of PrpA^CTD^ (55–86 residues) could mediate the change in the oligomerization states of PrpA^FL^ from tetramer to dimer, suggesting the importance of CTD for the PrpA^FL^ homotetramer. Furthermore, it seems that the homodimer is actually the minimal assembly unit of PrpA^2–54^ in the solution. Overall with the crystal structure of the PrpTA complex, it was certain that the PrpTA biological assembly unit indicated with PrpT:PrpA^2^:PrpT (Figure 1Ba) contains two PrpT monomers (Figure 1Bb), which were isolated from each other, and a PrpA homodimer (Figure 1Bd). In other words, PrpT:PrpA^2^:PrpT heterotetramer is maintained by one single PrpA^FL^ homodimer interface formed by N-terminal PrpA^RHH^ domains and two PrpT-PrpA^FL^ heterodimer interfaces, which is similar to its structurally relevant complex, such as *Cv*ParDE, and consistent with the conclusion that PrpA dimer interface and PrpT-PrpA heterodimer interface with an estimated CSS (Complex Formation Significance Score, calculated by PISA server) value of 1 contribute to PrpTA heterotetramer complexation. To sum up, the PrpTA complex exists as the PrpT:PrpA^2^:PrpT heterotetramer in solution and consists of two toxin monomers together with an antitoxin PrpA homodimer which appears to be depolymerized from homotetramer and the minimal functional unit of PrpA^FL^.

### Toxin PrpT belongs to the ParE family

**FIGURE 2:**
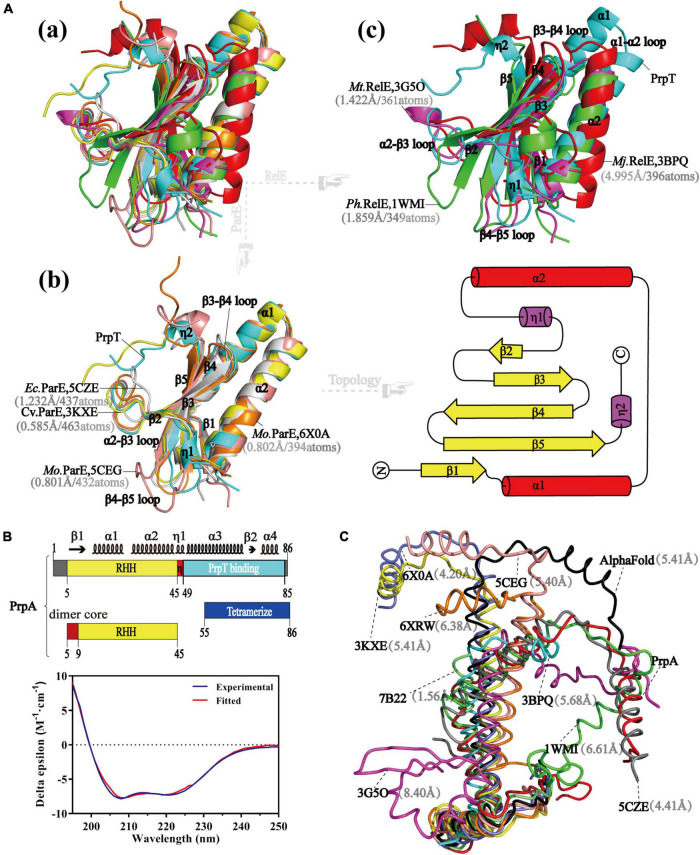
Structure of PrpT is conservative; however, PrpA has a novel structure. **(A)** The superposition of solved structures of relevant toxins **(a)**. The superposition of solved structures from group 1 with PrpT (left) and their topological structure diagram (right) **(b)**. The superposition of solved structures from group 2 and group 3 with PrpT **(c)**. **(B)** The architecture of PrpA^FL^ (upper) and corresponding CD spectrum (bottom). PrpA contains ∼47.9% helix, ∼0% sheet, ∼6.3% turn, and ∼45.7% unstructured fragments in solution. **(C)** The superposition of PrpA^FL^ with relevant antitoxin structures represented by ribbons. The structure of PrpA^FL^ is different from relevant antitoxin structures, especially the backbone track of CTD.

PrpT toxin starts from a RHH fragment in the N-terminus, helices of which are connected by a single glycine residue, and the β1 tends to be packed with the EF-P OB-like fold that is mainly composed of C-terminal 3-stranded antiparallel β sheet (β3–β5), as shown in Figure 1Bb. To determine accurately the protein family of PrpT, the sequence alignment, phylogenetic analysis, and structural superposition analysis with its homologs were performed. The sequence alignment and phylogenetic tree reflected that PrpT and its sequence homologs could be divided into three subgroups (Supplementary Figures 4A,B). In addition to the residue Leu that is highly conserved in the α 1 helix of all known structures, a conservative (Gln/Gly)-Gly diad is specific to members in group 1, that is, ParE family. Accordingly, the superposition of all solved structures of toxin proteins from the sequence alignment above could also be divided into three subgroups based on their structure deviations with toxin PrpT (Figure 2Aa). The crystal structure of PrpT is similar to structures of ParE family members with an average estimated r.m.s.d. of 0.855 Å and the main difference lies in the conformation of α 2–β3 and β4–β5 loops, which might play a key role in adjusting and maintaining the conservative three-dimensional structure (Figure 2Ab). However, structures from group 2 or group 3 could be distinguished apparently from PrpT due to large structure deviations mainly resulting from the flexibility of loops and the length differences of α 1 and α 2 with corresponding helixes in PrpT (Figure 2Ac). Therefore, the toxin PrpT could be assigned to the ParE family.

### PrpA^FL^ monomer structure has a novel three-dimensional folding

As illustrated in Figure 1Bc, PrpA^FL^ begins with an extended conformation (β1) spanning from 5 to 9 aa, followed by the α 1 helix (11–23 aa) and the α 2 helix (30–45 aa). The C-terminal PrpT neutralizing domain (49–85 aa), which is also the tetramerization domain, is connected with the C-terminus of the α 2 helix by a hinge region composed of AGS triad [Figure 2B (upper)]. Due to the presence of glycine residue in the N-terminus of α 1 helix, the centroidal axis is rotated ∼70° in relation to helix α 2. For a similar reason, the direction of the helix α 1 is actually rotated ∼80° with respect to the strand β1 (Supplementary Figure 5c). Compared to the ∼47.9% helix content of PrpA^FL^ in solution [Figure 2B (bottom)], the total helix content of PrpA^FL^ in the PrpTA complex is ∼55.7%. Higher helix content seems to hint at a conformational change occurring in the PrpA^CTD^ upon PrpT binding. The DALI topological analysis revealed not only the C-terminal diversity of CopG/MetJ/Arc family with the conservative RHH motif but also the structure uniqueness of PrpA^FL^. Despite the higher sequence similarity, PrpA^FL^ exhibits a difference in the secondary structural elements (α 2–α 3 region) with its homologs and obvious structural deviation in the backbone track of CTD (Figure 2C). Even though *Vc*ParD2 (PDB: 7B22) and *Ec*ParD (PDB: 5CZE) exhibit structural similarity to a different degree with the N-terminal PrpA^RHH^ domain (r.m.s.d. = 1.56 Å) and the PrpA^CTD^ (r.m.s.d. = 4.41 Å), respectively, the structure of PrpA^FL^ is still unique compared to structures already deposited in PDB together with the structure predicted by AlphaFold (r.m.s.d. = 5.41 Å). In summary, the folding of PrpA^FL^ is definitely novel and distinct from any other structures deposited in PDB.

### N-terminal 2-stranded antiparallel β-sheet located in ribbon–helix–helix domain is the oligomerization basis of PrpT:PrpA^2^:PrpT and PrpA

**FIGURE 3:**
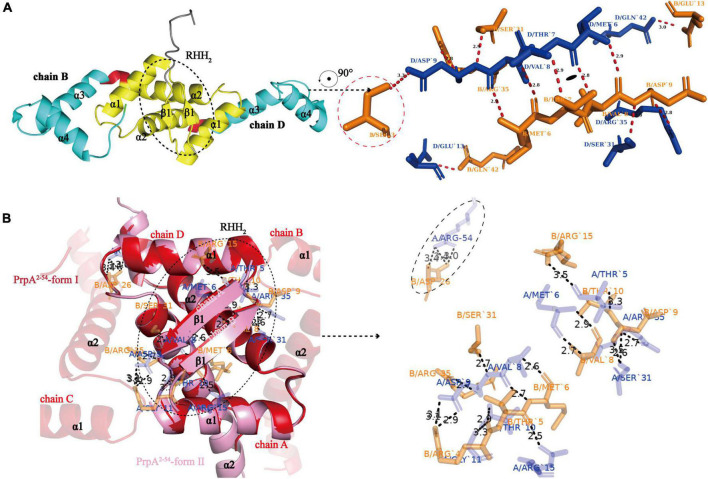
N-terminal 2-stranded antiparallel β-sheet formed by adjacent ribbons is the basis of oligomerization. **(A)** The cartoon representation of PrpA^FL^ dimer (left) and residues involved in the symmetric RHH dimer interface (right). The sole asymmetric Ser3B is enclosed with red dotted lines (Residues from chain B and chain D are highlighted in orange and blue sticks, respectively. The short red dashed lines indicate hydrogen bonds or salt bridges, and their matching distances are labeled in corresponding color). **(B)** PrpA^2–54^-form I and PrpA^2–54^-form II contain similar homodimer interface, and relevant residues of PrpA^2–54^-form II are shown as sticks and labeled in corresponding color. The only Arg54A-Asp26B contact between α 3 helixes of PrpA^2–54^ is enclosed with black-dotted lines, which appears to be caused by N-terhexahistidine and absent in PrpA^FL^ homodimer.

To investigate the structural details of PrpT:PrpA^2^:PrpT, crystal structures of the PrpTA complex and PrpA^2–54^ were all carefully analyzed. Like other members from the RHH family, PrpA monomer polymerizes in solutions and crystals in a highly symmetrical manner *via* a local twofold axis to form a two-stranded antiparallel β-sheet (Figure 3A). The symmetric dimerization interface could be found in PrpT:PrpA^2^:PrpT (excluding residue Ser3B) or either form of PrpA^2–54^ crystal structures (Figures 3A,B). Moreover, the comparative structural analysis highlighted that PrpA^2–54^ dimers are similar to each other with an r.m.s.d. value of ∼1.38 Å over 96 residues (Supplementary Figure 3). Thus, only the interface that existed in PrpA^2–54^-form II will be described in detail here. PISA analysis reflected that the hydrogen bond/salt bridge interaction network of dimerization interface is primarily offered by Met6, Val8, Asp9, Ser31, and Arg35 from each monomer together with Arg15A/B, Thr10A/B, Arg54A, Arg4B, and Asp26B for the PrpA^2–54^ (or symmetric Thr7A/B, Glu13A/B and asymmetric Ser3B for PrpA^FL^ in PrpTA complex). The dimerization interface in PrpTA or PrpA^2–54^-form II buries roughly 1700 Å^2^ with the nearly identical negative ΔG value (∼24 kcal/mol) corresponding to hydrophobic interfaces. It is presumably because bonds are broken by even small shifts in distance or orientation caused by the tag that interface symmetry of terminus that carries the tag of PrpA^FL^ or PrpA^2–54^ is always worse than that of the other one. In addition, a number of hydrophobic interactions between α helices could also partly contribute to the formation of a stable PrpA^FL^ homodimer. Crystal structures of PrpT:PrpA^2^:PrpT heterotetramer complex and PrpA^2–54^ suggested the significance of RHH dimer for the PrpT-PrpA recognition and the formation of PrpA^FL^ multi-dimer. Overall with LigPlus analysis (Supplementary Figure 6), it is rational to assert that the 2-stranded antiparallel β-sheet formed by two adjacent ribbons spanning from 5 to 9 aa is the basis of oligomerization of the PrpTA system.

### Flexibility-to-stability transition of PrpA^CTD^ upon the PrpT binding

**FIGURE 4:**
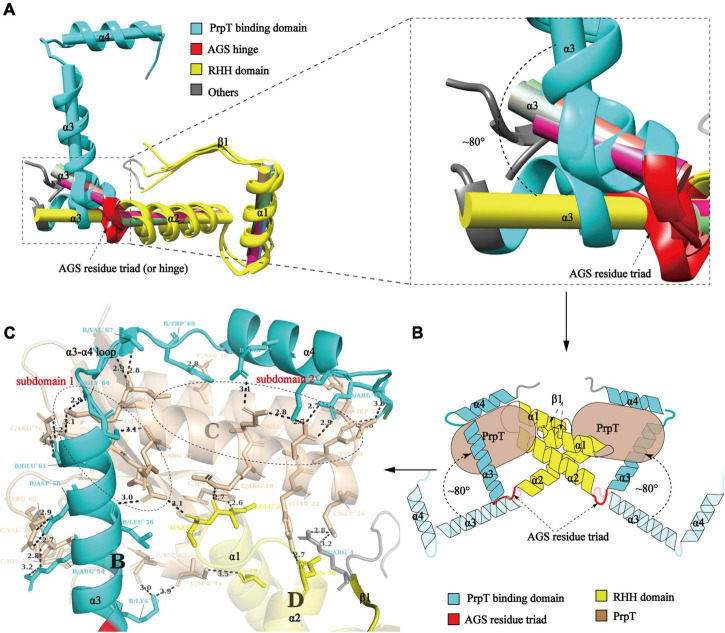
Conformational change in the PrpA^CTD^ triggered by toxin PrpT binding. **(A)** Cartoon representation of structural superposition of PrpA^FL^ monomer with PrpA^2–54^ monomers (left). The magnified version of α 3 helices is highlighted in the right panel (The centroid axis of each helix fragment is shown as cylinder utilizing UCSF Chimera software suite). **(B)** The proposed model of conformational change in the PrpA^CTD^ upon toxin PrpT binding. **(C)** The cartoon representation of the PrpT-PrpA heterodimer contact interface. Subdomains of PrpT are enclosed with black-dotted lines and labeled in red.

To investigate whether PrpA^CTD^ undergoes a conformational transition upon toxin PrpT binding, structural superposition of PrpA^2–54^ monomers with PrpA^FL^ was conducted. As demonstrated in Figure 4A, the results uncovered the obvious orientation change of α 3 upon PrpT binding, which is consistent with the flexibility feature of the PrpA^CTD^. Except for the asymmetric Arg54A-ASP26B contact in PrpA^2–54^, the protein backbones of PrpA^2–54^ or PrpA^FL^ project away from the dimer interface without further intra- or inter-contacts, suggesting the flexibility of PrpA^CTD^ (Figure 3B). That is, the PrpT binding could result in the dramatic flexibility-to-stability transition of PrpA^CTD^, which provokes the de-tetramerization of PrpA^FL^ antitoxin and the rotation of PrpA^CTD^ by ∼80° around the AGS residue triad, further leading to the extra interaction between toxin PrpT and RHH domains of PrpA homodimer (Figure 4B). PrpT:PrpA^2^:PrpT heterotetramer is formed *via* a local twofold axis and highlights two extremely similar PrpT-PrpA^FL^ contact interfaces. Therefore, only one heterodimer interface will be discussed here. PrpA and PrpT interact *via* two hydrophobic subdomains surrounded by electrostatic interactions (Supplementary Figures 5, 7). The α 3 and α 4 make up the core PrpT binding region, the surface polarities of which are exactly opposite to the two subdomains of PrpT, which are mainly comprised of N-terminal ribbon–helix-helix and the C-terminal 4-stranded anti-parallel β-sheet (β2–β5), respectively (Figure 4C). The heterodimer interface dominated by a series of salt bridges/hydrogen bonds covers a surface of ∼2000 Å^2^ with an estimated ΔG value of −17 kcal/mol. Even though the number of extra contacts between PrpT and PrpA^RHH^ accounts for just ∼20% of all PrpT-PrpA^FL^ interactions according to PISA, they are deemed to limit the swinging ability of PrpA^CTD^ in the local space, thus further enhancing the stability of PrpT:PrpA^2^:PrpT heterotetramer. Together, we infer that the conformational changes in the PrpA^CTD^ are significant for neutralizing the toxin.

**FIGURE 5:**
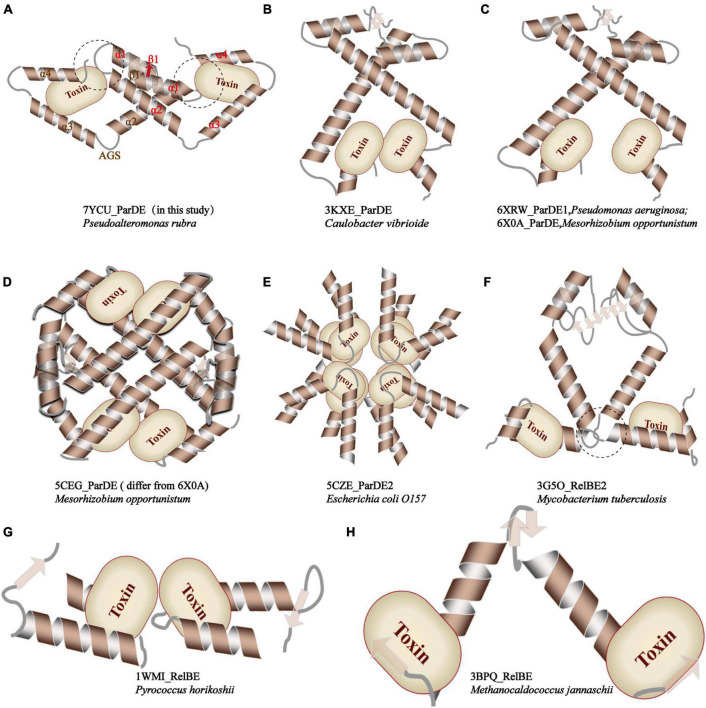
Comparison of the inter-molecular contact patterns in the toxin–antitoxin complexes with relevant structures. The PrpT toxin monomer in PrpT-PrpA^2^-PrpT heterotetramer could simultaneously interact with one CTD and both NTDs of the PrpA^FL^ homodimer after the CTDs undergo an obvious spatial change. This interaction mode is thought to enhance the stability of CTD of each PrpA^FL^ monomer by restricting its swinging ability in the local space and, therefore, further makes the heterotetramer much more stable neutralizing PrpT toxin.

Furthermore, compared with relevant toxin–antitoxin complexes reported before, as demonstrated in Figure 5, PrpT-PrpA^2^-PrpT heterotetramer is the only toxin–antitoxin complex whose toxin monomer could simultaneously interact with the CTD and the NTDs of the antitoxin homodimer, thus neutralizing the toxin. A similar strategy is also found in *Mt*RelBE2 (Figure 5F), of which adjacent α 2–α 3 loop could interact with each other limiting the flexibility of CTD, meanwhile, blocking the contacts between antitoxin NTD and toxin monomer. In brief, the PrpA dimerization interface, PrpA^CTD^-PrpT contacts, and PrpA^RHH^-PrpT contacts all contribute to PrpTA oligomerization. In actual fact, except for *Ec*ParDE (Figure 5E) and *Ph*RelBE (Figure 5G) whose oligomerization is maintained by toxin–toxin contacts that are actually absent in PrpT-PrpA^2^-PrpT heterotetramer, the oligomerization of *Cv*ParDE (Figure 5B), *Pa*ParDE (Figure 5C), *Mo*ParDE (Figure 5D), *Mt*RelBE (Figure 5F), and *Mj*RelBE (Figure 5H) all only depend on their NTDs, especially the N-terminal antiparallel β sheet. To sum up, the PrpT-PrpA^2^-PrpT heterotetramer displays a novel interaction profile between toxin and antitoxin, which contributes to the stability of the PrpTA complex.

### Dimer–dimer interface in PrpA^2–54^ multi-dimer possesses more extensive inter-monomer contacts

**FIGURE 6:**
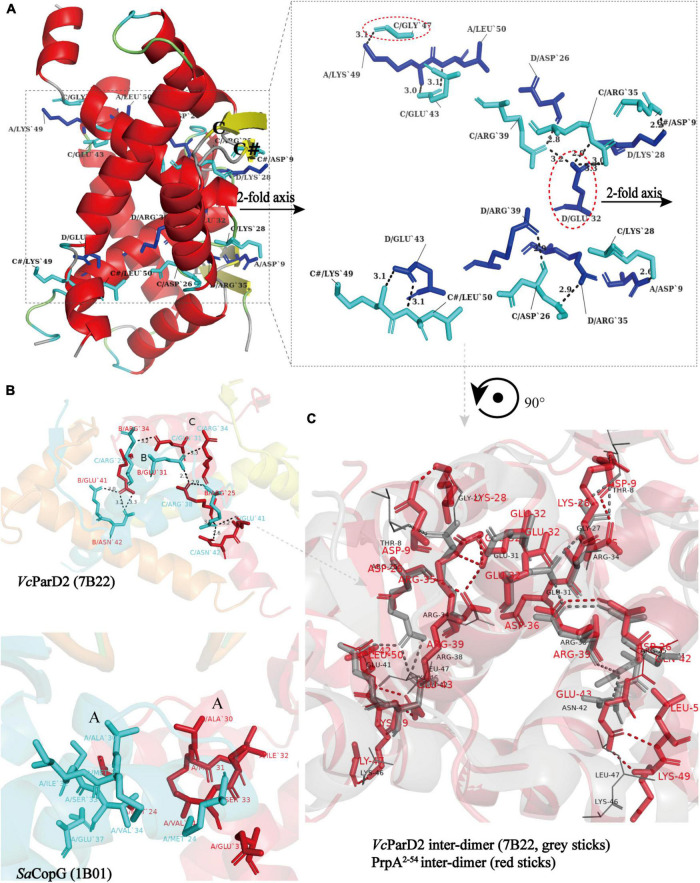
All monomers in PrpA^2–54^ homotetramer participate in tetramerization and have much more extensive contacts. **(A)** A view of the cartoon representation of the PrpA^2–54^ homotetramer, and the relevant residues in the dimer–dimer interaction interface are shown as cyan stick (chain C and C#) or blue stick (chain A and D) and labeled in black. The dimer–dimer interaction interface is magnified in the right panel (Hydrogen bonds or salt bridges are indicated by the short black-dotted lines and their distances labeled in black). **(B)** Cartoon representation of the *Vc*ParD2 tetramer (upper, PDB ID:7B22) and *Sa*CopG tetramer (bottom, PDB ID:1B01) (Residues involved in the inter-dimer interface are highlighted by the same color as the corresponding chain). **(C)** The superposition of inter-dimer interface of *Vc*ParD2 tetramer (gray sticks) and PrpA^2–54^ tetramer (red sticks) (Residues presented with lines in corresponding color actually do not participate in the tetramerization and are only used for comparison).

**FIGURE 7:**
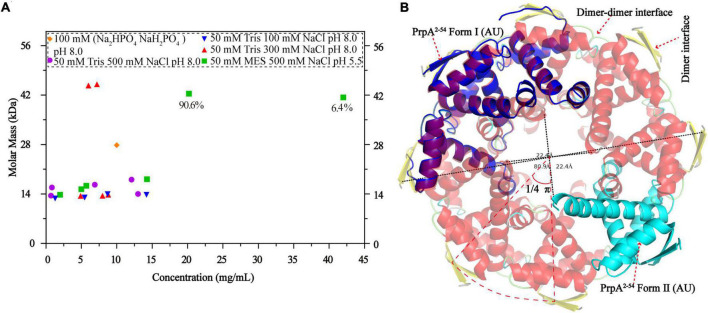
Phosphate buffer (PB) and the increase in protein concentration contribute to PrpA^2–54^ oligomerization. **(A)** Size exclusion chromatography- multi-angle light scattering experiments (SEC-MALS) assays are conducted in Tris–HCl buffers with different pH and ion strength. **(B)** Two forms of PrpA^2–54^ could assembly into a higher-order hexadecamer in crystal. The maximum diameters of a PrpA^2–54^ hexadecamer and the hole located in the center are ∼80.9 and ∼22.4Å, respectively. The 2-stranded antiparallel β-sheets of adjacent PrpA^2–54^ homodimers produce an arc of ∼π/4. The ability of RHH motif dimerization and the highly symmetric inter-dimer interface is the basis of the formation of multi-dimer unit.

Similar to the arrangement of CopG (PDB: 2CPG) (Gomis-Ruth et al., 1998; Costa et al., 2001) monomers in the asymmetric unit (AU), PrpA^2–54^-form I monomers are arranged in a cogwheel-like form in the AU [Supplementary Figure 3 (right)]. Apart from a type A-D dimer, either B or C could further dimerize with its symmetric mate generating type B-B# and type C-C# dimers (# represents symmetry mate). Since the dimer–dimer interfaces in AD-BB# and AD-CC# are similar to each other with an overall r.m.s.d. of 1.05 over 144 residues, only the AD-CC# interface will be, thus, discussed at length here. As demonstrated in Figure 6A (left), a functional PrpA^2–54^ tetramer is defined by inter-dimer crystallographic contacts between RHH domains with positively charged DNA-binding surfaces exposed to the solvent environment. The AD and CC# interact *via* a surface that is electrostatically complementary and somewhat slightly hydrophobic, especially the interface formed by α 2 helixes of PrpA. Salt bridges and hydrogen bonds below 4 Å are distributed mainly through the α 1–α 2 turn and α 2 helices of molecule AD together with almost equivalent parts of a molecule CC# [Figure 6A (right)]. In addition, the interface generated by AD and CC# dimers covers a total surface area of ∼1100 Å^2^, which is a little smaller than that of the dimerization interface. The inter-dimer interface of PrpA^2–54^ is thought to be strong enough since it can still exist as a multi-dimer in certain cases in the absence of PrpA^CTD^ (Figure 7A). A similar dimer–dimer interface dominated by hydrogen bonds and salt bridges was also reported in *Vc*ParD2 tetramer [Figure 6B (upper)], especially the symmetric residues E32, R35, and R39 (matching E31, R34, and R38 in *Sa*CopG, respectively) are extremely conserved in primary and tertiary structures. However, they are all totally different with inter-dimer interface dominated by hydrophobic van de Waals interaction that existed in *Sa*CopG tetramer bound to DNA [Figure 6B (bottom)]. However, the inter-dimer interface of PrpA^2–54^ displays a novel feature that all monomers could participate in the formation of tetramer suggesting a much more extensive and strong contact. In contrast, few residues from one single monomer from each dimer, that is, only two chains mediate the corresponding inter-dimer interface for *Sa*CopG and *Vc*ParD2. The difference in the inter-dimer interface presumably lies in the different conformations caused by residue substitutions, such as Asp9, Glu43, and Lys28 (corresponding to Thr8, Gly27, and Asn42 in *Vc*ParD, respectively), which allows much more complex contacts between PrpA^2–54^ monomers (Figure 6C). Moreover, an unfavorable positive–positive interaction is abolished by Gly47 substitution matching the Lys46 in *Vc*ParD. In general, the dimer–dimer interface in PrpA^2–54^ multi-dimer possesses much more extensive inter-monomer contacts.

### An increase in the protein concentration and phosphate buffer contributes to the PrpA^2–54^ oligomerization

A circular hexadecamer (∼112.58 kDa) consisting of 8 PrpA^2–54^ dimers could be found in both forms of PrpA^2–54^ crystals (Figure 7B). Higher-order form of PrpA^2–54^ is maintained by a dimer interface and inter-dimer interface. The maximum diameter of the annular doughnut-like hexadecamer is ∼80 Å and that of the hole in the center is approximately 22 Å. The C-terminal α 2 helix of each PrpA^2–54^ monomer stretches outward from the inner side of the hexadecamer; overall with the flexibility of CTD, it is reasonable to infer that the replacement of PrpA^FL^ would provide an entropic penalty for oligomerization, which is similar to the effect made by the IDR of *Vc*ParD2. To further investigate which factors could mediate the oligomerization of PrpA^2–54^, a series of SEC-MALS experiments were performed (Figure 7A). PrpA^2–54^ tends to present an absolute molecular weight matching PrpA^2–54^ dimer in most cases, and no clear dependency on the ion strength or pH of solutions could be observed. However, the increase in protein concentration within a limited range seems to result in a higher absolute molecular weight (41.38–42.41 kDa, probably hexamer). In addition, the hexadecamer could be occasionally observed in solution with an extremely smaller proportion (∼4%). The PrpA^2–54^ has a tendency to assemble into a relatively stable homotetramer in phosphate buffer (PB), which is distinct from its performance in Tris–HCl buffers. Taken together, the increase in the protein concentration and PB are favorable for the oligomerization of PrpA^2–54^ in the solution.

## Conclusion and discussion

Toxins from the ParE/RelE family, PrpT included, share higher sequence and structural similarities with one another (Garcia-Rodriguez et al., 2021a; Klemencic et al., 2021; Ni et al., 2021; Zhou et al., 2021a). In contrast, antitoxins from the RHH superfamily could be different from one another in sequence with diverse CTD conformations (Raumann et al., 1994; Costa et al., 2001; Weihofen et al., 2006; Schreiter and Drennan, 2007). The antitoxin PrpA^FL^ with an extremely conservative N-terminal RHH motif exists as a homotetramer in solution; however, PrpA^FL^ tends to exist as the minimal functional homodimer other than homotetramer in the absence of PrpA^CTD^. The symmetric homodimer interface and the inter-dimer interface with much more complicated inter-monomer contacts mediate PrpA^2–54^ homotetramer and homohexamer in solution and appear to be a potential prerequisite for assembling into a circular hexadecamer. PB and the increase in concentration will have a small impact on the oligomerization of PrpA^2–54^. In addition, comparative analysis of PrpA^FL^ with PrpA^2–54^ monomers in different oligomeric states reflected that the PrpA^CTD^ is relatively flexible, which is presumably concerned mainly with the inconsecutive α 2–α 3 helix and little contacts between CTDs of PrpA^FL^ homodimer. However, the relatively flexible PrpA^CTD^ could become stable upon PrpT binding, which might be involved in the extra contacts between toxin PrpT and PrpA^RHH^ domains caused by the conformational change in PrpA^CTD^.

**FIGURE 8:**
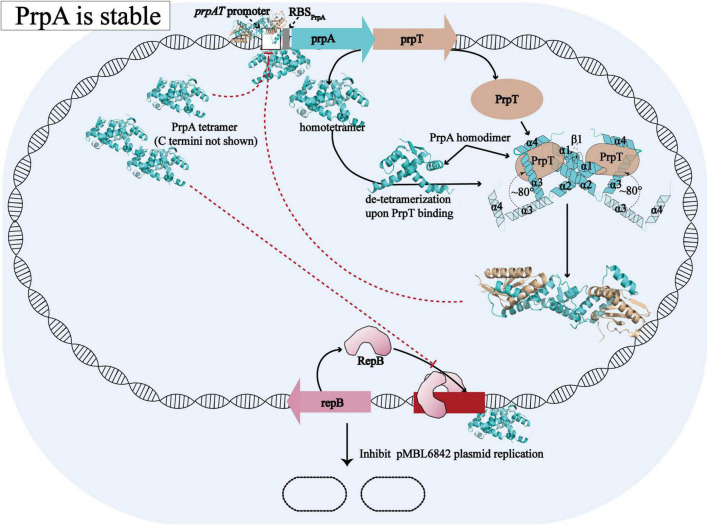
Structural insights into the molecular mechanism underlying how PrpTA TAS controls plasmid replication. The schematic diagram presented above is drawn based on the model proposed by Ni et al. (2021).

Overall with the structural evidence in our study, the model proposed before for the molecular mechanism [proposed by Ni et al. (2021)] underlying how PrpT/PrpA system controls plasmid replication could be further improved and the updated schematic diagram is shown in Figure 8. When PrpA is intact, antitoxin PrpA tends to exist as a functional homotetramer defined by the PrpA^RHH^-PrpA^RHH^ contacts and unknown PrpA^CTD^-PrpA^CTD^ contacts between PrpA^FL^ homodimer. The flexible CTDs of PrpA^FL^ are supposed to swing in a small local space, especially those in PrpA^FL^ homodimer. After the PrpA^CTD^ comes across the cognate toxin PrpT, the PrpA^FL^ homotetramer depolymerized into two isolated homodimers establishing strong interactions with toxin PrpT monomers, which results in the formation of PrpT:PrpA^2^:PrpT heterotetramer and the neutralization of PrpT toxicity. In other words, the PrpA^FL^ homodimer tends to neutralize toxin PrpT in preference to binding to another PrpA^FL^ homodimer, and the homodimer seems to be the most unstable form due to free CTDs. The binding of PrpT to the PrpA^CTD^ actually results in an obvious conformational change, rotating the PrpA^CTD^ nearly 80° in relation to AGS triad to establish extra PrpT-PrpA^RHH^ mutual interactions, further stabilizing PrpT:PrpA^2^:PrpT heterotetramer. Meanwhile, PrpA homotetramer alone and PrpT:PrpA^2^:PrpT heterotetramer could bind to the *prpAT* operon, resulting in the transcriptional inhibition of the PrpTA module. In addition, PrpA could also competitively bind to the iteron sequences in the *ori*, interfering with the binding of replication initiator RepB to the *ori* site, thus preventing the overreplication of the plasmid. In contrast, PrpA is degraded during the stress condition, thus abolishing the inhibition of the RepB binding to *ori* (Ni et al., 2021).

**FIGURE 9:**
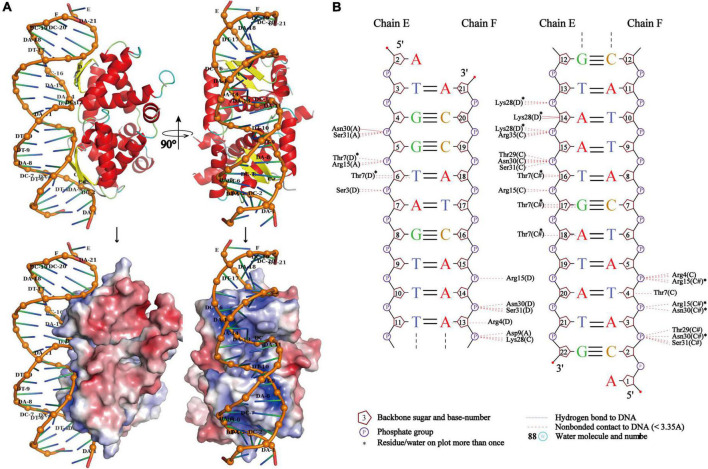
PrpA^2–54^ homotetramer binds to duplex *prpAT* promoter with the N-terminal 2-stranded antiparallel β-sheets docking into the major grooves of DNA. **(A)** The side view of the cartoon representation of PrpA^2–54^ homotetramer-*prpAT* promoter (upper left) and its corresponding electrostatic potential surface [red, negative and blue positive (bottom left)]. The top view of PrpA^2–54^ homotetramer-*prpAT* model (upper right), and its corresponding surface (bottom right). **(B)** The schematic presentation of the interaction pattern existed between PrpA^2–54^ homotetramer and *prpAT* promoter (NUCPLOT, version: 1.0).

Due to the high symmetry of the PrpA homodimer interface and inter-dimer interface, and structural features of members from the RHH family, multiple multi-dimeric (in complex with dsDNA) structures of PrpA^2–54^ could be predicted. In this study, a model of PrpA^2–54^ in complex with promoter dsDNA was modeled to investigate possible interaction profiles between PrpA^2–54^ and dsDNA. The model highlighted that antiparallel β-sheets of PrpA^2–54^ tetramer could establish interactions with the duplex *prpAT* promoter [Figure 9A (upper)]. Sequence-specific and non-specific contacts could be mediated mainly by β-sheets and helices, respectively, with N-terminal, the 2-stranded antiparallel β-sheet of PrpA^2–54^ docking favorably into the major grooves of *prpAT* promoter, thus inducing dsDNA to bend obviously to interact better with positively charged DNA-binding surfaces of PrpA^2–54^ [Figures 9A (bottom),B]. Compared to an ideal type B dsDNA, the negatively charged phosphodiester backbone of the promoter bent more than 30° due to compression of both major and minor grooves facing the tetramer. Bent dsDNA accounts for the base pairs adjacent to the center of the promoter being somewhat inclined; however, the phosphodiester backbone of the promoter excluding the central part still stretches along a flat track. In contrast, TFs such as CopG, Arc, and MetJ could bend the minimal cognate operator with two repressor binding sites by 40° to 60°, which is a bit higher than that of PrpA^2–54^ and the discrepancy seems to result mainly from the varying spacer between DNA-binding sites (Raumann et al., 1994; Gomis-Ruth et al., 1998; Garvie and Phillips, 2000; Weihofen et al., 2006). In addition, CopG and MetJ could recognize the pseudo- or palindromic sites, however, Arc does not. Another thing is that most of the sidechains situated in the corresponding positions of the β-sheets in Arc, MetJ, and CopG make contacts with different operator base positions (Raumann et al., 1994), which indicates that the N-terminal β-sheet is the DNA-binding recognition element, such as the second α helix of HTH motif. Although PrpA and Arc all undergo conformational changes, it is the PrpA^CTD^ that would suffer dramatic conformational changes upon the corepressor binding, which is not the same case with Arc whose N-terminal β-sheets do undergo conformational changes. These results are consistent with the conclusion that the RHH superfamily could recognize and bind nucleotides in the major grooves of duplex DNA by changing the conformation of β-sheet, sugar-phosphodiester backbone track, and sequence identity together with fine-tuned sidechains to build specific or unspecific contacts (Raumann et al., 1994; Gomis-Ruth et al., 1998; Costa et al., 2001; Schreiter and Drennan, 2007). However, due to the flexibility in PrpA^CTD^, it is difficult to accurately predict the model of PrpA^FL^ homotetramer in complex with duplex DNA. Based on our structural insights into the PrpTA system, we speculated that CTDs in PrpA^FL^ homotetramer appear to feature with (a) solvent-exposed PrpT binding surface; (b) weaker contact interface than PrpA^RHH^-PrpA^RHH^ interface and PrpT-PrpA interface; and (c) some flexibility to arrest toxin PrpT. The interaction mode found in DNA-bound TraM (PDB: 3ON0) from *Escherichia coli* appears to satisfy all hypotheses mentioned above. In summary, our results are supposed to contribute to elucidating the PrpTA complex assembly mechanism, protein oligomerization, and the mechanism underlying how PrpTA TAS controls plasmid replication, which may help to understand the emergence of drug-resistant bacteria and MDT *via* a similar mechanism.

## Data availability statement

The data presented in this study are deposited in the Protein Data Bank (PDB) repository, and the accession numbers are 7YCU, 7YCV, and 7YCW (https://www.rcsb.org/).

## Author contributions

LN, ZZ, and CW conceived the idea and designed the frame of this study. CW and CN performed the experiments and analyzed the data. CW wrote the manuscript. KH and LX revised the manuscript. All authors contributed to the article and approved the submitted version.

## References

[B1] Bertelsen M. B. Senissar M. Nielsen M. H. Bisiak F. Cunha M. V. Molinaro A. L. (2021). Structural basis for toxin inhibition in the VapXD toxin-antitoxin system. *Structure* 29 139–150.e3. 10.1016/j.str.2020.10.002 33096014 PMC7867571

[B2] Blair J. M. Webber M. A. Baylay A. J. Ogbolu D. O. Piddock L. J. (2015). Molecular mechanisms of antibiotic resistance. *Nat. Rev. Microbiol.* 13 42–51. 25435309 10.1038/nrmicro3380

[B3] Bobay B. G. Andreeva A. Mueller G. A. Cavanagh J. Murzin A. G. (2005). Revised structure of the AbrB N-terminal domain unifies a diverse superfamily of putative DNA-binding proteins. *FEBS Lett.* 579 5669–5674. 10.1016/j.febslet.2005.09.045 16223496

[B4] Cohen N. R. Lobritz M. A. Collins J. J. (2013). Microbial persistence and the road to drug resistance. *Cell Host Microbe* 13 632–642. 23768488 10.1016/j.chom.2013.05.009 PMC3695397

[B5] Coles M. Djuranovic S. Soding J. Frickey T. Koretke K. Truffault V. (2005). AbrB-like transcription factors assume a swapped hairpin fold that is evolutionarily related to double-psi beta barrels. *Structure* 13 919–928. 10.1016/j.str.2005.03.017 15939023

[B6] Cooper T. F. Heinemann J. A. (2000). Postsegregational killing does not increase plasmid stability but acts to mediate the exclusion of competing plasmids. *Proc. Natl. Acad. Sci. U.S.A.* 97 12643–12648. 11058151 10.1073/pnas.220077897 PMC18817

[B7] Costa M. Sola M. Del Solar G. Eritja R. Hernández-Arriaga A. M. Espinosa M. (2001). Plasmid transcriptional repressor CopG oligomerises to render helical superstructures unbound and in complexes with oligonucleotides. *J. Mol. Biol.* 310 403–417. 10.1006/jmbi.2001.4760 11428897

[B8] De Bruyn P. Girardin Y. Loris R. (2021). Prokaryote toxin–antitoxin modules: Complex regulation of an unclear function. *Protein Sci.* 30 1103–1113. 33786944 10.1002/pro.4071 PMC8138530

[B9] Del Solar G. H. De Al Campa A. G. Perez-Martin J. Choli T. Espinosa M. (1989). Purification and characterization of RepA, a protein involved in the copy number control of plasmid pLS1. *Nucleic Acids Res.* 17 2405–2420. 10.1093/nar/17.7.2405 2497439 PMC317632

[B10] Du C. Zhang W. Gu H. Dong X. Hu Y. (2022). Type II toxin–antitoxin system, RatAB, contributes to oxidative resistance, biofilm formation and virulence of Edwardsiella piscicida. *Aquacult. Res.* 53 2575–2585. 10.3389/fmicb.2021.646299 33732226 PMC7957083

[B11] Edelmann D. Oberpaul M. SCHäBERLE T. F. Berghoff B. A. (2020). Post-transcriptional deregulation of the tisB/istR-1 toxin–antitoxin system promotes SOS-independent persister formation in *Escherichia coli*. *Environ. Microbiol. Rep.* 13 159–168. 10.1111/1758-2229.12919 33350069

[B12] Fleming A. (1929). On the antibacterial action of cultures of a penicillium, with special reference to their use in the isolation of B. influenzæ. *Br. J. Exp. Pathol.* 10 226–236. 11545337

[B13] Fraikin N. Goormaghtigh F. Van Melderen L. (2020). Type II toxin-antitoxin systems: Evolution and revolutions. *J Bacteriol* 202:e00763-19. 10.1128/JB.00763-19 31932311 PMC7167474

[B14] Garcia-Rodriguez G. Girardin Y. Volkov A. N. Singh R. K. Muruganandam G. Van Dyck J. (2021b). Entropic pressure controls the oligomerization of the *Vibrio cholerae* ParD2 antitoxin. *Acta Crystallogr. D Biol. Crystallogr.* 77(Pt 7) 904–920. 10.1107/S2059798321004873 34196617 PMC8251345

[B15] Garcia-Rodriguez G. Girardin Y. Singh R. K. Volkov A. N. Konijnenberg A. Sobott F. (2021a). *Vibrio cholerae* ParE2 toxin modulates its operon transcription by stabilization of an antitoxin DNA ruler. *bioRxiv* [Preprint] 10.1101/2021.03.22.436508

[B16] Garvie C. W. Phillips S. E. (2000). Direct and indirect readout in mutant Met repressor-operator complexes. *Structure* 8 905–914. 10.1016/s0969-2126(00)00182-9 10986458

[B17] Gerdes K. Rasmussen P. B. Molin S. (1986). Unique type of plasmid maintenance function: Postsegregational killing of plasmid-free cells. *Proc. Natl. Acad. Sci. U.S.A.* 83 3116–3120. 3517851 10.1073/pnas.83.10.3116 PMC323463

[B18] Gomis-Ruth F. X. Sola M. Acebo P. Párraga A. Guasch A. Eritja R. (1998). The structure of plasmid-encoded transcriptional repressor CopG unliganded and bound to its operator. *EMBO J.* 17 7404–7415. 10.1093/emboj/17.24.7404 9857196 PMC1171085

[B19] Hayes F. Van Melderen L. (2011). Toxins-antitoxins: Diversity, evolution and function. *Crit. Rev. Biochem. Mol. Biol.* 46 386–408. 21819231 10.3109/10409238.2011.600437

[B20] Huemer M. Mairpady Shambat S. Brugger S. D. Zinkernagel A. S. (2020). Antibiotic resistance and persistence-Implications for human health and treatment perspectives. *EMBO Rep.* 21:e51034. 10.15252/embr.202051034 33400359 PMC7726816

[B21] Jaffe A. Ogura T. Hiraga S. (1985). Effects of the ccd function of the F plasmid on bacterial growth. *J. Bacteriol.* 163 841–849. 10.1128/jb.163.3.841-849.1985 3897195 PMC219208

[B22] Jurenas D. Fraikin N. Goormaghtigh F. Van Melderen L. (2022). Biology and evolution of bacterial toxin-antitoxin systems. *Nat. Rev. Microbiol.* 20 335–350. 34975154 10.1038/s41579-021-00661-1

[B23] Kamruzzaman M. Wu A. Y. Iredell J. R. (2021). Biological functions of type II toxin-antitoxin systems in bacteria. *Microorganisms* 9:1276. 10.3390/microorganisms9061276 PMC8230891 34208120

[B24] Kim J.-S. Wood T. K. (2016). Persistent persister misperceptions. *Front. Microbiol.* 7 2134. 10.3389/fmicb.2016.02134 PMC5187198 28082974

[B25] Klemencic M. Haluzan Vasle A. Dolinar M. (2021). The cysteine protease MaOC1, a prokaryotic caspase homolog, cleaves the antitoxin of a type II toxin-antitoxin system. *Front. Microbiol.* 12:635684. 10.3389/fmicb.2021.635684 33679669 PMC7935541

[B26] Knight K. L. Sauer R. T. (1989). DNA binding specificity of the Arc and Mnt repressors is determined by a short region of N-terminal residues. *Proc. Natl. Acad. Sci. U.S.A.* 86 797–801. 10.1073/pnas.86.3.797 2644643 PMC286564

[B27] Lee K. Y. Lee B. J. (2016). Structure, biology, and therapeutic application of toxin-antitoxin systems in pathogenic bacteria. *Toxins* 8:305. 10.3390/toxins8100305 27782085 PMC5086665

[B28] Leroux M. Culviner P. H. Liu Y. J. Littlehale M. L. Laub M. T. (2020). Stress can induce transcription of toxin-antitoxin systems without activating toxin. *Mol. Cell* 79:280–292.e8. 32533919 10.1016/j.molcel.2020.05.028 PMC7368831

[B29] Levin-Reisman I. Ronin I. Gefen O. Braniss I. Shoresh N. Balaban N. Q. (2017). Antibiotic tolerance facilitates the evolution of resistance. *Science (New York, NY)* 355 826–830. 10.1126/science.aaj2191 28183996

[B30] Li B. Wang P. Zeng Z. Cai X. Wang G. Wang X. (2016). Complete genome sequence of *Pseudoalteromonas rubra* SCSIO 6842, harboring a putative conjugative plasmid pMBL6842. *J. Biotechnol.* 224 66–67. 10.1016/j.jbiotec.2016.03.010 26970053

[B31] Li M. Gong L. Cheng F. Yu H. Zhao D. Wang R. (2021). Toxin-antitoxin RNA pairs safeguard CRISPR-Cas systems. *Science (New York, NY)* 372:eabe5601. 10.1126/science.abe5601 33926924

[B32] Ma D. Gu H. Shi Y. Huang H. Sun D. Hu Y. (2021). *Edwardsiella piscicida* YefM-YoeB: A type II toxin-antitoxin system that is related to antibiotic resistance, biofilm formation, serum survival, and host infection. *Front. Microbiol.* 12:646299. 10.3389/fmicb.2021.646299 33732226 PMC7957083

[B33] Maisonneuve E. Gerdes K. (2014). Molecular mechanisms underlying bacterial persisters. *Cell* 157 539–548. 24766804 10.1016/j.cell.2014.02.050

[B34] Muthuramalingam M. White J. C. Bourne C. R. (2016). Toxin-antitoxin modules are pliable switches activated by multiple protease pathways. *Toxins* 8:214. 10.3390/toxins8070214 27409636 PMC4963847

[B35] Ni S. Li B. Tang K. Yao J. Wood T. K. Wang P. (2021). Conjugative plasmid-encoded toxin-antitoxin system PrpT/PrpA directly controls plasmid copy number. *Proc. Natl. Acad. Sci. U.S.A.* 118:e2011577118. 10.1073/pnas.2011577118 33483419 PMC7848731

[B36] Ogura T. Hiraga S. (1983). Mini-F plasmid genes that couple host cell division to plasmid proliferation. *Proc. Natl. Acad. Sci. U.S.A.* 80 4784–4788. 6308648 10.1073/pnas.80.15.4784 PMC384129

[B37] Page R. Peti W. (2016). Toxin-antitoxin systems in bacterial growth arrest and persistence. *Nat. Chem. Biol.* 12 208–214. 26991085 10.1038/nchembio.2044

[B38] Paul P. Patel P. Verma S. K. Mishra P. Sahu B. R. Panda P. K. (2022). The Hha–TomB toxin–antitoxin module in *Salmonella enterica* serovar Typhimurium limits its intracellular survival profile and regulates host immune response. *Cell Biol. Toxicol.* 38 111–127. 10.1007/s10565-021-09587-z 33651227

[B39] Paul P. Sahu B. R. Suar M. (2019). Plausible role of bacterial toxin-antitoxin system in persister cell formation and elimination. *Mol. Oral Microbiol.* 34 97–107. 10.1111/omi.12258 30891951

[B40] Qi X. Brothers K. M. Ma D. Mandell J. B. Donegan N. P. Cheung A. L. (2021). The *Staphylococcus aureus* toxin–antitoxin system YefM–YoeB is associated with antibiotic tolerance and extracellular dependent biofilm formation. *J. Bone Joint Infect.* 6 241–253. 10.5194/jbji-6-241-2021 34262845 PMC8273624

[B41] Raumann B. E. Rould M. A. Pabo C. O. Sauer R. T. (1994). DNA recognition by beta-sheets in the Arc repressor-operator crystal structure. *Nature* 367 754–757. 8107872 10.1038/367754a0

[B42] Sarpong D. D. Murphy E. R. (2021). RNA regulated toxin-antitoxin systems in pathogenic bacteria. *Front. Cell Infect. Microbiol.* 11:661026. 10.3389/fcimb.2021.661026 PMC8167048 34084755

[B43] Schreiter E. R. Drennan C. L. (2007). Ribbon-helix-helix transcription factors: Variations on a theme. *Nat. Rev. Microbiol.* 5 710–720. 10.1038/nrmicro1717 17676053

[B44] Schumacher M. A. Balani P. Min J. Chinnam N. B. Hansen S. Vuliæ M. (2015). HipBA-promoter structures reveal the basis of heritable multidrug tolerance. *Nature* 524 59–64. 10.1038/nature14662 26222023 PMC7502270

[B45] Schumacher M. A. Min J. Link T. M. Guan Z. Xu W. Ahn Y. H. (2012). Role of unusual P loop ejection and autophosphorylation in HipA-mediated persistence and multidrug tolerance. *Cell Rep.* 2 518–525. 10.1016/j.celrep.2012.08.013 22999936 PMC4831868

[B46] Solar G. D. Hernández-Arriaga A. M. Gomis-Rüth F. X. Coll M. Espinosa M. (2002). A genetically economical family of plasmid-encoded transcriptional repressors involved in control of plasmid copy number. *J. Bacteriol.* 184 4943–4951. 10.1128/JB.184.18.4943-4951.2002 12193609 PMC135303

[B47] Song S. Wood T. K. (2020). Toxin/Antitoxin system paradigms: Toxins bound to antitoxins are not likely activated by preferential antitoxin degradation. *Adv. Biosyst.* 4:e1900290. 10.1002/adbi.201900290 32293143

[B48] Srivastava A. Pati S. Kaushik H. Singh S. Garg L. C. (2021). Toxin-antitoxin systems and their medical applications: Current status and future perspective. *Appl. Microbiol. Biotechnol.* 105 1803–1821. 10.1007/s00253-021-11134-z 33582835

[B49] Tharappel A. M. Li Z. Li H. (2022). Inteins as drug targets and therapeutic tools. *Front Mol Biosci* 9 821146. 10.3389/fmolb.2022.821146 PMC8861304 35211511

[B50] Van Melderen L. (2010). Toxin-antitoxin systems: Why so many, what for? [J]. *Curr. Opin. Microbiol.* 13 781–785. 21041110 10.1016/j.mib.2010.10.006

[B51] Weihofen W. A. Cicek A. Pratto F. Alonso J. C. Saenger W. (2006). Structures of omega repressors bound to direct and inverted DNA repeats explain modulation of transcription. *Nucleic Acids Res.* 34 1450–1458. 10.1093/nar/gkl015 16528102 PMC1401508

[B52] Xia K. Ma J. Liang X. (2021). Impacts of type II toxin-antitoxin systems on cell physiology and environmental behavior in acetic acid bacteria. *Appl. Microbiol. Biotechnol* 105 4357–4367. 10.1007/s00253-021-11357-0 34021811

[B53] Xie Y. Wei Y. Shen Y. Li X. Zhou H. Tai C. (2018). TADB 2.0: An updated database of bacterial type II toxin-antitoxin loci. *Nucleic Acids Res.* 46 D749–D753. 10.1093/nar/gkx1033 29106666 PMC5753263

[B54] Xue L. Khan M. H. Yue J. Zhu Z. Niu L. (2022). The two paralogous copies of the YoeB-YefM toxin-antitoxin module in *Staphylococcus aureus* differ in DNA binding and recognition patterns. *J. Biol. Chem.* 298 101457. 10.1016/j.jbc.2021.101457 34861238 PMC8717551

[B55] Zhang S. P. Feng H. Z. Wang Q. Kempher M. L. Quan S. W. Tao X. (2021). Bacterial type II toxin-antitoxin systems acting through post-translational modifications. *Comput. Struct. Biotechnol. J.* 19 86–93. 10.1016/j.csbj.2020.12.002 33384857 PMC7758455

[B56] Zhou J. Li S. Li H. Jin Y. Bai F. Cheng Z. (2021b). Identification of a toxin–antitoxin system that contributes to persister formation by reducing NAD in *Pseudomonas aeruginosa*. *Microorganisms* 9:753. 10.3390/microorganisms9040753 33918483 PMC8065639

[B57] Zhou J. Du X. J. Liu Y. Gao Z. Q. Geng Z. Dong Y. H. (2021a). Insights into the neutralization and DNA binding of toxin-antitoxin system ParE^SO^-CopA^SO^ by structure-function studies. *Microorganisms* 9:2506. 10.3390/microorganisms9122506 34946107 PMC8706911

