# Supplementary material for: Structural insights into the PrpTA toxin–antitoxin system in Pseudoalteromonas rubra
Source: Front Microbiol. 2022 Nov 24;13:1053255. doi: 10.3389/fmicb.2022.1053255 (PMC9731233; doi:10.3389/fmicb.2022.1053255)
Supplement: Supplementary file 1 [file Data_Sheet_1.docx]

**Supplementary Table S1**

| Protein | Synthesis Vector ID | Sequence |
| --- | --- | --- |
| PrpA^FL^ | PrpA-N-his-pET28a | MGSSHHHHHHSSGLVPRGSHMSSRTMTVDTGEELRAFVEGLVESGDYKTNSEVIRDGLRLLQEKTAGSKLAALRQLIDEGEQSGEAVPWDRDSFLARMRQKGPRGG |
| PrpA^2-54^ | PrpA^2-54^-C-his-pET28a) | MGSSRTMTVDTGEELRAFVEGLVESGDYKTNSEVIRDGLRLLQEKTAGSKLAALRLEHHHHHH |
| PrpT | PrpT-pET22b(+) | MVVANTLVLKPRAEQDLERIFEYSYTEFGWQQAQQYISDLDQTFQTLAASTDLAINYDHVRPGLKAFPVGAHIVFFRATDTGIEVIRVLHQSMDYPRHV |

* AGS triad is colored in red.

**Supplementary Table S2**

| **Data collection and Refinement** | **PrpA^2-54^-form Ⅰ** | **PrpA^2-54^-form Ⅱ** | **PrpTA** |
| --- | --- | --- | --- |
| Beamlilne | SSRF-BL19U1 | SSRF-BL02U1 | SSRF-BL02U1 |
| Wavelength (Å) | 0.9791 | 0.9792 | 0.9792 |
| Resolution range (Å) | 30.08-2.2 | 30.34-2.612 | 27.02-1.793 |
|  | (2.279-2.2) | (2.706-2.612) | (1.857-1.793) |
| Space group | *P*4_2_2_1_2 | *I*422 | *P*2_1_ |
| Unit cell a b c (Å) | 61.837 61.837 129.974 | 83.252 83.252 70.8 | 44.782 83.085 56.489 |
| α β γ (°) | 90 90 90 | 90 90 90 | 90 91.659 90 |
| Total reflections | 323803 (33438) | 54655 (4979) | 258103 (21203) |
| Unique reflections | 13454 (1294) | 3976 (373) | 38256 (3738) |
| Multiplicity | 24.1 (25.8) | 13.7 (13.3) | 6.7 (5.7) |
| Completeness (%) | 99.71 (98.92) | 99.25 (97.90) | 98.64 (97.22) |
| Mean I/sigma(I) | 30.41 (5.95) | 16.46 (2.99) | 16.70 (2.90) |
| Wilson B-factor (Å^2^) | 43.46 | 62.7 | 21.43 |
| *R_merge_* | 0.06929 (0.601) | 0.1038 (0.8511) | 0.08416 (0.7913) |
| *R_meas_* | 0.07089 (0.613) | 0.108 (0.8837) | 0.0912 (0.8716) |
| *R_pim_* | 0.01471 (0.1194) | 0.02865 (0.2309) | 0.03478 (0.3589) |
| CC_1/2_ | 0.999 (0.989) | 0.998 (0.907) | 0.999 (0.809) |
| CC* | 1 (0.997) | 1 (0.975) | 1 (0.946) |
| Reflections used in refinement | 13453 (1285) | 3976 (373) | 38253 (3738) |
| Reflections used for *R_free_* | 664 (58) | 188 (19) | 1919 (205) |
| *R_work_* | 0.2188 (0.3241) | 0.2276 (0.3170) | 0.1675 (0.2309) |
| *R_free_* | 0.2498 (0.3517) | 0.2714 (0.4428) | 0.2000 (0.2832) |
| CC_work_ | 0.965 (0.848) | 0.968 (0.883) | 0.959 (0.817) |
| CC_free_ | 0.946 (0.725) | 0.972 (0.838) | 0.951 (0.772) |
| Number of non-hydrogen atoms | 1674 | 849 | 3242 |
| macromolecules | 1608 | 846 | 2888 |
| solvent | 66 | 3 | 354 |
| Protein residues | 210 | 109 | 362 |
| R.M.S.deviation (bonds) (Å) | 0.008 | 0.012 | 0.01 |
| R.M.S.deviation (angles) (°) | 0.96 | 1.44 | 1.41 |
| Ramachandran favored (%) | 99.5 | 98.1 | 98.87 |
| Ramachandran allowed (%) | 0.5 | 1.9 | 1.13 |
| Ramachandran outliers (%) | 0 | 0 | 0 |
| Rotamer outliers (%) | 0 | 1.1 | 0 |
| Clashscore | 3.4 | 12.35 | 3.33 |
| Average B-factor (Å^2^) | 52.42 | 62.88 | 25.5 |
| macromolecules | 52.46 | 62.89 | 24.44 |
| solvent | 51.4 | 58.69 | 34.08 |

* Values in parentheses are for the outer shell.


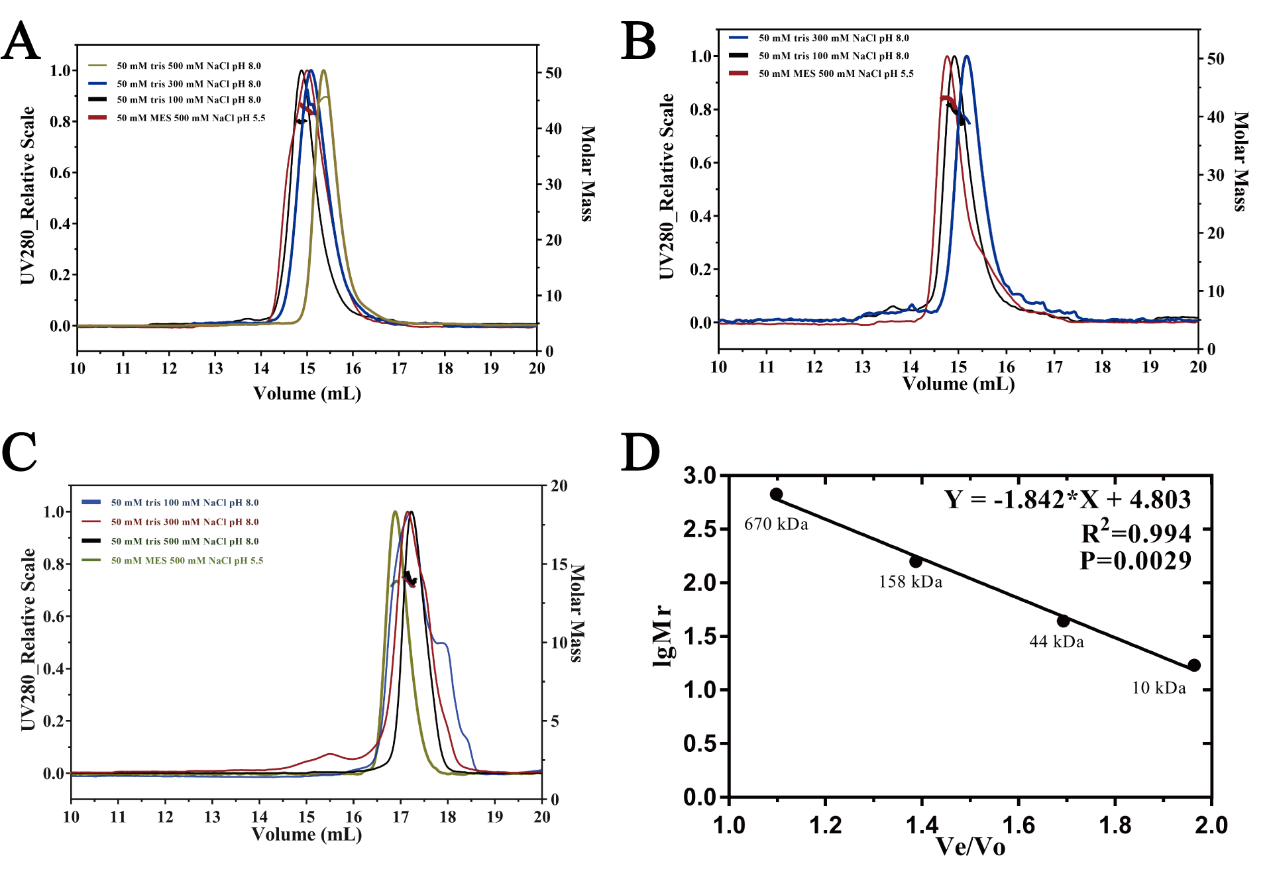


**Supplementary Figure S1 |** Oligomeric states of PrpTA, PrpA and PrpA^2-54^ based on SEC-MALS experiments. SEC-MALS reflected an estimated mass of 43.36 (±1.31) kDa for PrpTA **(A)**, 41.14 (±1.62) kDa for PrpA^FL^ **(B)** and 13.90 (±0.10) kDa for PrpA^2-54^ **(C)**, suggesting that PrpTA, PrpA^FL^ and PrpA^2-54^ exists as heterotetramer, homotetramer and homodimer, respectively, in solution. According to the standard curve of Superdex^TM^ 200 Increase 10/300 GL chromatographic column, the relative molecular weights of PrpTA and PrpA is 36.20 (±1.96) kDa and 36.74(±3.70) kDa, respectively **(D)**, which are basically consistent with the results generated by SEC-MALS.


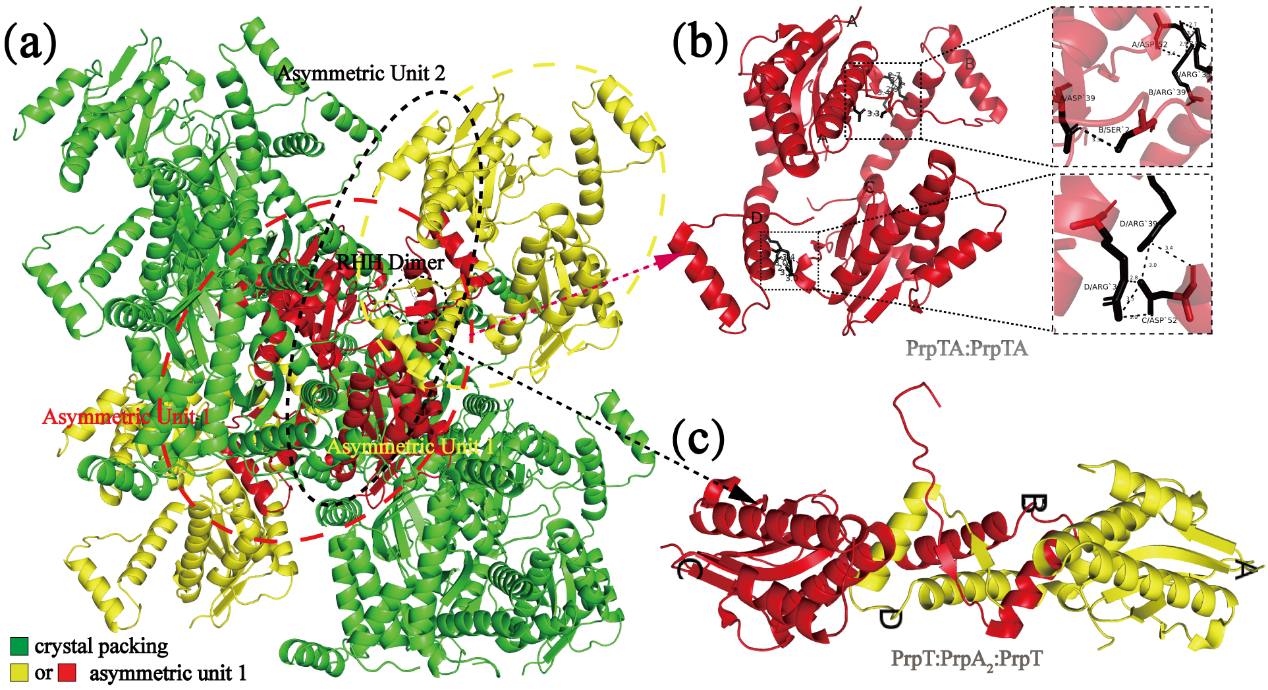


**Supplementary Figure S2 |** PrpTA exists as the PrpT:PrpA_2_:PrpT heterotetramer in solution. Cartoon representation of the stacking of unit cells in the PrpTA crystal (**a**) and its two kinds of asymmetric units indicated with PrpTA:PrpTA (**b**) and PrpT:PrpA_2_:PrpT (**c**), respectively. Compared to PrpT:PrpA_2_:PrpT, PrpTA:PrpTA contains less polar contacts mediated by Asp39A-Ser2B, Asp52A-Arg35B, Asp52A-Arg39B, Asp52C-Arg39D and Asp52C-Arg35D. According to PISA server, an estimated CSS value of 1 for the PrpA dimer interface or PrpT-PrpA heterodimer interface suggests that these two interfaces contribute greatly to PrpTA complexation.


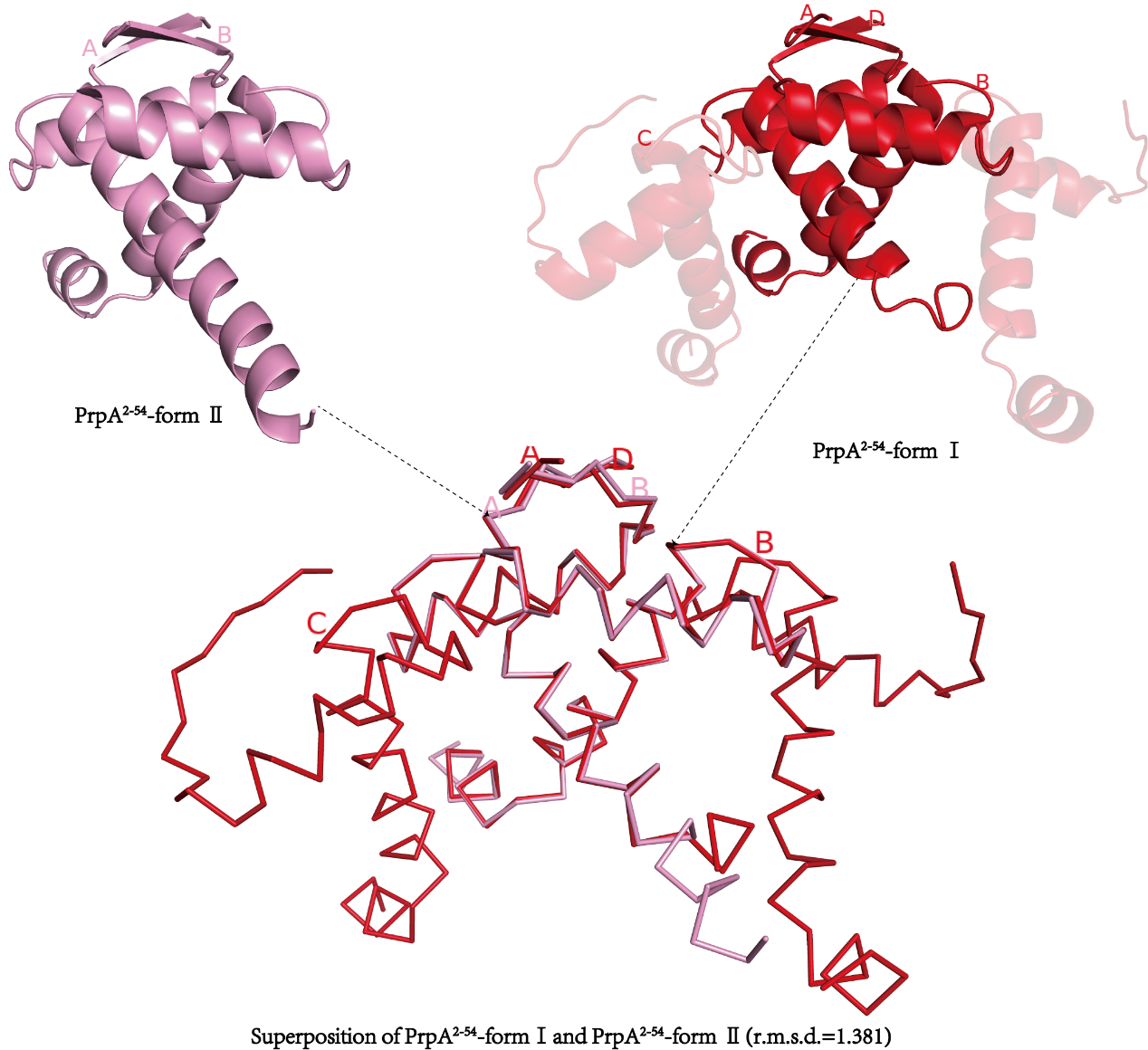


**Supplementary Figure S3 |** PrpA^2-54^-form Ⅰ (red) and PrpA^2-54^-form Ⅱ (pink) both contain a homodimer with a r.m.s.d. value of 1.381 Å over 96 residues. The ribbon presentation (**bottom**) of the superposition of PrpA^2-54^-form Ⅰ (**upper left**) with PrpA^2-54^-form Ⅱ (**upper right**), and similar homodimer interfaces exist in 2 forms of crystal structures of PrpA^2-54^.


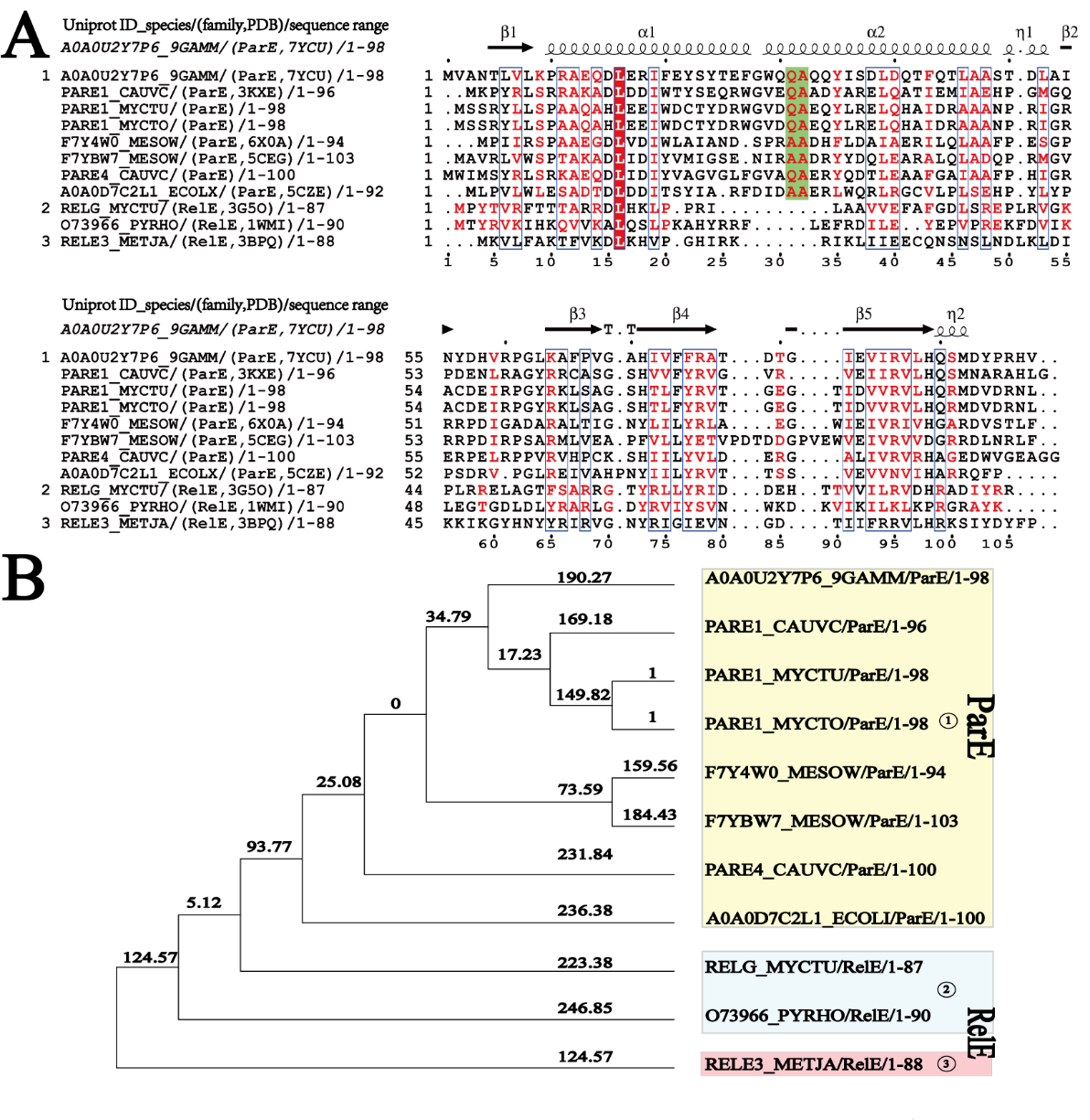


**Supplementary Figure S4** | PrpT belongs to ParE family. **(A)** The alignment of PrpT with homologous toxin sequences using ESPript server (version 3.0) and the secondary elements of PrpT indicated on the top are defined by DSSP plugin. (The strictly conserved amino acid residues and those similar in a group are colored white with red box and red, respectively. Residues that are different amongst conserved groups and those similar across groups are enclosed by green fluo box and the blue frame, respectively.) **(B)** Phylogenetic analysis of toxin proteins is based on **Figure A** utilizing Neighbor-Joining method in Jalview (version: 2.11.2.3).


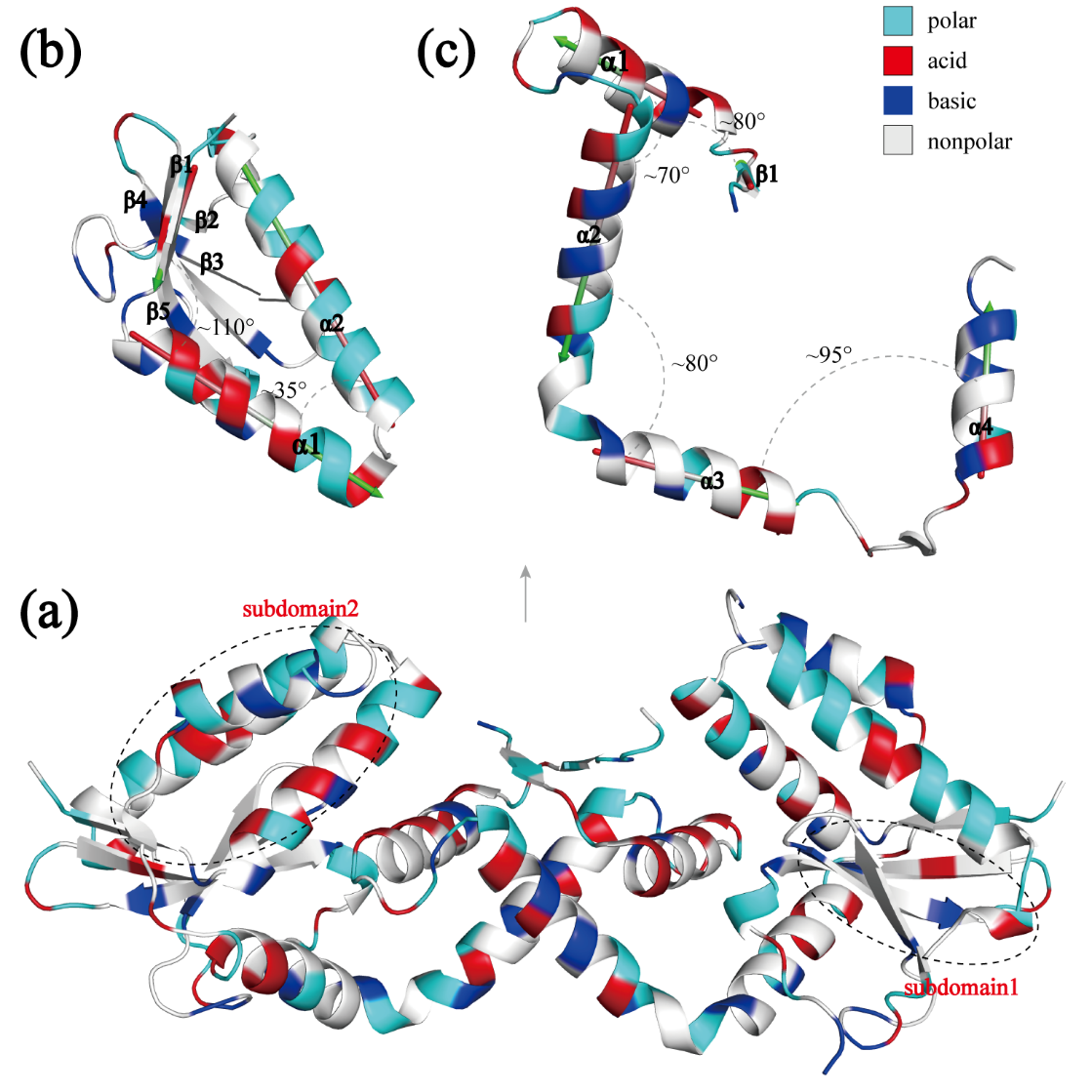


**Supplementary Figure S5 |** Features on residue distribution of PrpT and PrpA in PrpT:PrpA_2_:PrpT heterotetramer. Polar residues located in PrpT subdomain 2 with a formal charge of -4 tend to be distributed on the side facing PrpA^FL^ α4, however, nonpolar residues tend to be distributed on the surface facing PrpA^FL^ α3. The nonpolar residues situated in α1, α3, α4 helixes of PrpA^FL^ are all basically distributed on the side facing toxin PrpT, and those from α2 are all found on the side facing the other monomer. In addition, polar residues from α1 and α4 of PrpA tend to interact with polar residues from N-terminal helixes of PrpT (**a**). Angles between secondary elements including α helixes and β-strand are indicated in PrpT (**b**) and PrpA (**c**).


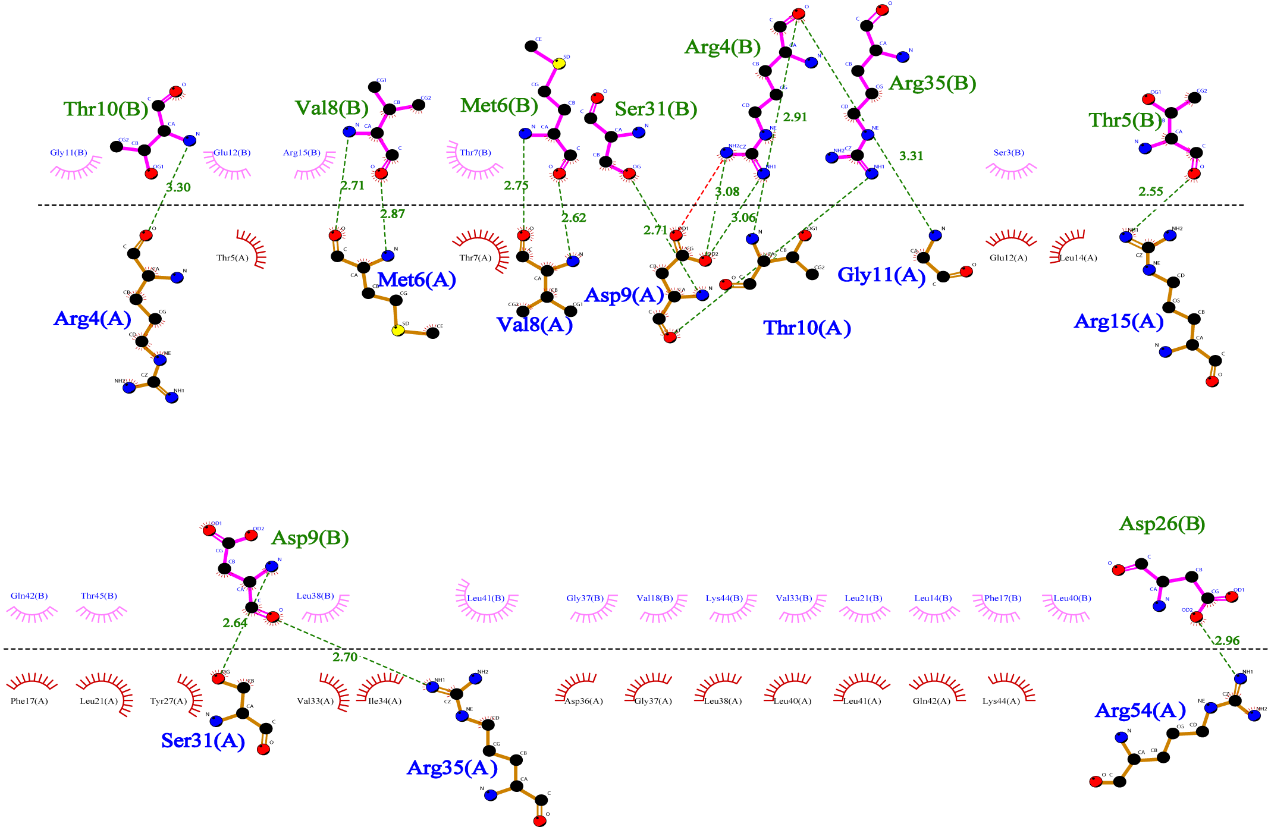


**Supplementary Figure S6 |** Polar bonds existed in N-terminal 2-stranded antiparallel β-sheet of PrpA^2-54^ homodimer contribute mostly to the dimerization. Polar bonds are almost distributed in or near the interface formed by N-terminal ribbons, and hydrophobic interactions are mainly found between α2 helixes. (Hydrogen bonds and salt bridges are indicated by green and red dotted lines, respectively, with distance labelled in green. Pink or red arcs with short lines on the outer side stand for hydrophobic interaction (by LigPlus)).


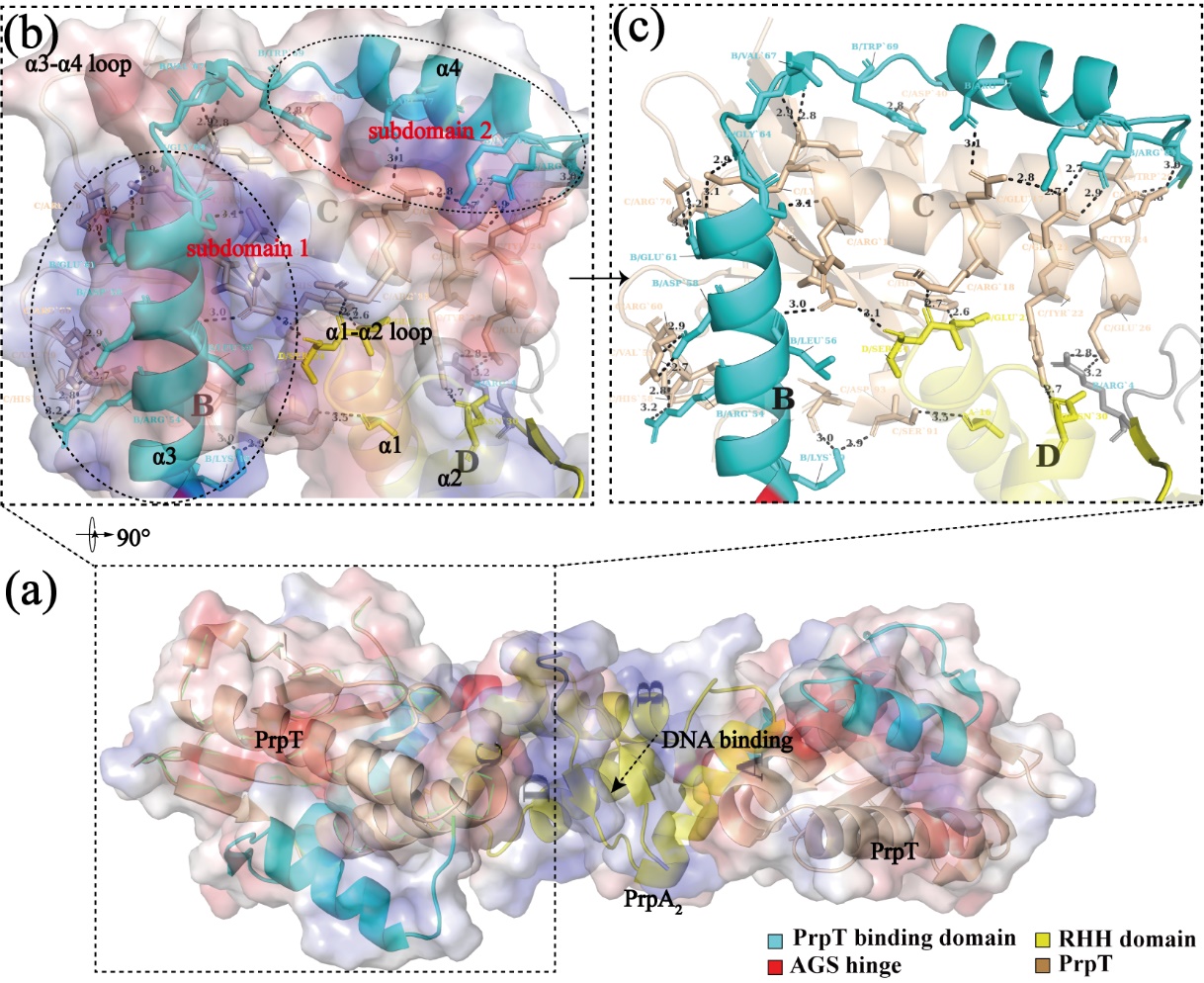


**Supplementary Figure S7 |** PrpA and PrpT interact via two hydrophobic patches surrounded by a number of electrostatic interactions. A view of the PrpT-PrpA_2_-PrpT heterotetramer cartoon representation (**bottom**) and its corresponding electrostatic potential surface generated by APBS (red, negative and blue positive). And The PrpT-PrpA contact interface is enlarged in figures (**a**) and (**b**), and the relevant residues are presented by wheat (PrpT; Chain C), cyan and yellow sticks (PrpA; Chain B and D, respectively), respectively. Two subdomains of PrpT toxin are enclosed by black dotted lines and labelled in figure (**a**), which interact with α3 and α4 of PrpA antitoxin, respectively.
